# Chloroplast Z-ring dynamics is governed by conserved core regions of evolutionarily divergent FtsZs

**DOI:** 10.3389/fpls.2025.1622675

**Published:** 2025-07-30

**Authors:** Lingyan Cao, Katie J. Porter, Wenbin Du, Emily J. Tallerday, Moyang Liu, Wanqi Liang, Katherine W. Osteryoung, Cheng Chen

**Affiliations:** ^1^ Joint International Research Laboratory of Metabolic and Developmental Sciences, State Key Laboratory of Hybrid Rice, School of Life Sciences and Biotechnology, Shanghai Jiao Tong University, Shanghai, China; ^2^ Department of Plant Biology, Michigan State University, East Lansing, MI, United States; ^3^ Department of Plant Sciences, School of Agriculture and Biology, Shanghai Jiao Tong University, Shanghai, China; ^4^ Shanghai Collaborative Innovation Center of Agri-Seeds, Shanghai Jiao Tong University, Shanghai, China; ^5^ Joint Center for Single Cell Biology, Shanghai Jiao Tong University, Shanghai, China

**Keywords:** chloroplast, FtsZ-ring, dynamics, division, evolution

## Abstract

The chloroplast FtsZ ring (Z ring) is assembled by two distinct FtsZ proteins, FtsZ2 and FtsZ1 (referred to as FtsZA and FtsZB in red algae). FtsZ2 confers stability to the Z ring, while FtsZ1 enhances its dynamics. Enhanced Z-ring dynamics is essential for Z-ring remodeling, which drives chloroplast constriction and division. However, the mechanisms underlying the distinct dynamic properties of the two FtsZs remain unclear. Here, we report that the conserved core regions are primarily responsible for the distinct dynamic properties observed in both plant and red algal FtsZs. We demonstrate that the conserved core region of FtsZ1 enhances the dynamics of FtsZ2 within coassembled filaments. Likewise, we show that the conserved core region of red algal FtsZB promotes the dynamics of coassembled FtsZA rings. Our findings provide evidence that the evolution of a second FtsZ protein represents a general mechanism to enhance the dynamics of the chloroplast Z ring.

## Introduction

Chloroplasts originated through endosymbiosis when a free-living cyanobacterium was engulfed by a non-photosynthetic eukaryote approximately one billion years ago. The engulfed cyanobacterium gradually evolved to become a permanent organelle within the host cells (Gould et al., 2008; Sibbald and Archibald, 2020). Similar to their prokaryotic ancestors, chloroplasts are propagated through binary fission (division in the middle), ensuring their accurate inheritance during cytokinesis. Chloroplast division is orchestrated by a combination of proteins derived from both cyanobacteria and the host eukaryotes (Maple et al., 2005; TerBush et al., 2013; Osteryoung and Pyke, 2014; Chen et al., 2018). Among them, the cytoskeletal protein Filamentous temperature-sensitive Z (FtsZ) plays a central role, analogous to its function in bacterial cell division (Erickson et al., 2010).

The prokaryotic FtsZ is a tubulin-like GTPase that can self-assemble in the presence of GTP (Erickson, 1997). This protein is highly conserved across a range of species, from bacteria to algae and plants (Margolin, 2005; TerBush et al., 2013; McQuillen and Xiao, 2020). Each FtsZ harbors a conserved core region, which includes the GTP binding and GTPase activation domains, respectively, flanked by less conserved N- and C-terminal regions (**Supplementary Figure S1**). GTP-bound FtsZ monomers polymerize head to tail to form filamentous structures known as protofilaments (Oliva et al., 2004). The GTPase active site is formed longitudinally at the interface between adjacent FtsZ subunits (Scheffers et al., 2002). Upon GTP hydrolysis at the active site, the interface between two FtsZ subunits becomes less stable in the absence of GTP, leading to the disassociation of the GDP-bound subunit from the protofilament. This disassociated GDP-bound subunit is then recycled back into the soluble pool of FtsZ monomers, where it can be reactivated by binding to GTP (Mingorance et al., 2001). Consequently, the protofilaments exhibit dynamic behavior, effectively treadmilling at a steady state (Bisson-Filho et al., 2017; Yang et al., 2017; Corbin and Erickson, 2020). In bacteria, FtsZ protofilaments overlap to form a ring-like structure known as the FtsZ ring (Z ring) in the middle of the cell (Bi and Lutkenhaus, 1991; Du and Lutkenhaus, 2019; McQuillen and Xiao, 2020). The Z ring is anchored to the plasma membrane through interaction with membrane proteins and serves as a scaffold to recruit additional division proteins to form a mature divisome (Du and Lutkenhaus, 2019). The dynamics of the FtsZ protofilaments is critical for the constriction of the Z ring and thus for bacterial cell division *in vivo* (Redick et al., 2005; Osawa and Erickson, 2011; McQuillen and Xiao, 2020).

Unlike the bacterial Z ring, which is assembled from a single type of FtsZ, the chloroplast Z ring in the model organism *Arabidopsis thaliana* is composed of two distinct FtsZ proteins, AtFtsZ1 and AtFtsZ2 (Osteryoung and Vierling, 1995; Osteryoung et al., 1998; Vitha et al., 2001; Stokes and Osteryoung, 2003; TerBush et al., 2013). Both proteins are encoded by nuclear genes and are targeted to the chloroplasts via their N-terminal transit peptides (Osteryoung and Vierling, 1995; McAndrew et al., 2001), which are removed upon import into the chloroplast stroma, thus not being part of the mature protein. Studies have shown that AtFtsZ1 and AtFtsZ2 function non-redundantly and are both essential for the establishment of the chloroplast Z ring and for chloroplast division (Osteryoung et al., 1998; Schmitz et al., 2009). Immunolocalization studies have revealed that AtFtsZ1 and AtFtsZ2 colocalize to the chloroplast Z ring in both wild-type plants and numerous chloroplast division mutants (McAndrew et al., 2001; Vitha et al., 2001; Zhang et al., 2013). Additionally, they have been demonstrated to coassemble both *in vitro* (Olson et al., 2010) and *ex vivo* (TerBush and Osteryoung, 2012; TerBush et al., 2016; Yoshida et al., 2016; Chen et al., 2019). Phylogenetic analysis suggests that AtFtsZ2 originated from prokaryotic organisms, while AtFtsZ1 likely arose from the duplication of AtFtsZ2 (TerBush et al., 2013, 2018). AtFtsZ2 bears a conserved C-terminal peptide (CTP), a motif also found in bacterial FtsZs. Tethering of the chloroplast Z ring to the inner envelop membrane is primarily achieved through the interaction of the AtFtsZ2 CTP with the chloroplast membrane protein ARC6 (Maple et al., 2005). In contrast to AtFtsZ2, AtFtsZ1 lacks a defined CTP and does not interact with any known chloroplast membrane proteins, suggesting that the CTP may represent an important functional difference between the two FtsZs (Vitha et al., 2003; Maple et al., 2005; TerBush et al., 2013). Interestingly, recent studies have reported that AtFtsZ1 can directly associate with the inner envelop membrane through its C-terminal amphiphilic motif (Liu et al., 2022; An et al., 2024), indicating that AtFtsZ1 contributes to the tethering of the chloroplast Z ring *in vivo* as well.

Like the bacterial Z ring, the chloroplast Z ring is thought to be highly dynamic, although its substructure is not fully understood. Chloroplast Z-ring dynamics is challenging to study *in planta* due to their incorporation into a macromolecular complex with the rest of division machinery. Consequently, heterologous yeast systems, such as *Schizosaccharomyces pombe* (fission yeast) and *Pichia pastoris* (budding yeast), have been employed to probe the assembly and dynamics of chloroplast FtsZ filaments or rings (TerBush and Osteryoung, 2012; TerBush et al., 2016; Yoshida et al., 2016; Sung et al., 2018; TerBush et al., 2018; Chen et al., 2019; Porter et al., 2023). These yeast models offer a cellular environment, and their lack of endogenous FtsZ or other chloroplast division regulators makes them ideal for *ex vivo* investigation of the assembly and dynamic behaviors of the chloroplast FtsZ filaments and rings. Both AtFtsZ1 and AtFtsZ2 form homopolymerized filaments when expressed individually in *S. pombe* (TerBush and Osteryoung, 2012; TerBush et al., 2016). Coexpression analysis has revealed that AtFtsZ2 filaments exhibit slower turnover dynamics (subunit exchange) compared to AtFtsZ1, and that AtFtsZ1 can accelerate AtFtsZ2 dynamics in coassembled filaments (TerBush and Osteryoung, 2012; TerBush et al., 2018). Moreover, AtFtsZ1 and AtFtsZ2 heteropolymerize into a ring that can constrict in *Pichia pastoris*, with constriction correlating with increased dynamics of the reconstituted chloroplast Z ring (Yoshida et al., 2016). Overall, AtFtsZ2 appears to confer stability to the chloroplast Z ring, while AtFtsZ1 enhances its dynamics (TerBush et al., 2013). However, the underlying mechanisms governing the distinct dynamic properties of these two FtsZs remain largely elusive.

Rhodophyta (the lineage of red algae) diverged from the common ancestors of Viridiplantae (the lineage of green algae and land plants) and Glaucophyta (Strassert et al., 2021). In red algae, two FtsZ proteins, FtsZA and FtsZB, have been identified to be involved in chloroplast division (Miyagishima et al., 2004). FtsZA, similar to its green lineage counterpart FtsZ2, also possesses a CTP. However, it remains unclear whether the anchoring of the Z ring to the chloroplast membrane in red algae is mediated by interactions between the FtsZA CTP and membrane proteins. In contrast, FtsZB lacks a defined CTP and is believed to have duplicated from FtsZA, making it similar to FtsZ1 (Miyagishima et al., 2004). Both FtsZA and FtsZB are GTPases with conserved core regions flanked by less conserved C- and N-terminal regions, a structural feature common to FtsZ proteins across species. The core regions of FtsZA and FtsZB from the red alga *Galdieria sulphuraria* (Gs) have been shown to assemble *in vitro*, with coassembly promoting enhanced disassembly dynamics (Chen et al., 2017), a phenomenon not observed in the coassembly of AtFtsZ2 and AtFtsZ1 *in vitro* (Porter et al., 2021). These observations suggest that red algal FtsZs may possess unique assembly and dynamic properties. Notably, phylogenetic analysis indicates that the duplication of FtsZs in red algae occurred after the divergence of the red and green lineage (Stokes and Osteryoung, 2003; Miyagishima et al., 2011), suggesting that the evolution of a second FtsZ may be critical for chloroplast division in both lineages.

Previously, we assessed the biochemical behaviors of AtFtsZs using purified proteins and found that AtFtsZ2 assembled into protofilaments *in vitro*, whereas AtFtsZ1 did not (Porter et al., 2021). When coexpressed, AtFtsZ1 restrained the assembly of AtFtsZ2 protofilaments. We also discovered that the conserved core regions of these proteins largely recapitulated these biochemical features (Porter et al., 2021). However, a critical limitation of these *in vitro* assays, including those with GsFtsZs (Chen et al., 2017), is the limited ability to draw conclusions about the dynamic turnover of FtsZ filaments or rings. To delve into the mechanisms determining the distinct dynamic properties of the two chloroplast FtsZs, we utilized *P. pastoris* to examine the assembly and dynamics of chloroplast FtsZ filaments and rings. By directly measuring and comparing the turnover dynamics of Z rings reconstituted from both full-length and conserved core regions of FtsZs, we demonstrate that the conserved core regions predominantly govern the distinct dynamic properties of the two FtsZs in both plants and red algae. Furthermore, our study provides evidence that the evolution of a second FtsZ is a general mechanism to enhance the dynamics of the chloroplast Z ring.

## Materials and methods

### Construction of plasmids

All expression vector constructions were performed using the Gibson assembly method (Gibson et al., 2009). We prepared the backbone vectors from previously utilized single expression vectors, pPICZ A-AtFtsZ1_FL_-mCerulean and pPICZ A-AtFtsZ2_FL_-eYFP-MTS (Yoshida et al., 2016). These were then digested with EcoRI/XbaI or EcoRI/KpnI to excise the *AtFtsZ1_FL_* or *AtFtsZ2_FL_* fragment. For expression of the full-length FtsZ proteins in *Pichia*, the predicted transit peptides were excluded: AtFtsZ2 and AtFtsZ1 (1–48 aa and 1–57 aa, respectively), GsFtsZA and GsFtsZB (1–52 aa and 1–57 aa, respectively). The conserved core regions (CCRs) for FtsZs were defined as follows: AtFtsZ2_C_ (119–424 aa), AtFtsZ1_C_ (74–377 aa), GsFtsZA_C_ (121–424 aa), and GsFtsZB_C_ (91–404 aa). All the primers used in this study were listed in **Supplementary Table S3**.

To generate *G. sulphuraria* FtsZA and FtsZB constructs, synthesized *GsFtsZA* and *GsFtsZB* sequences, which were codon-optimized for *A. thaliana* for better comparison to their *Arabidopsis* counterparts (TerBush et al., 2018), were used as templates. Full-length *GsFtsZs* were amplified using primer sets LY33/LY34 and LY242/LY244, while the *CCRs* were amplified using primer sets LY36/LY37 and LY243/LY246. The obtained fragments were inserted into the EcoRI/XbaI digested pPICZ A-mCerulean backbone. Similarly, the *CCRs* of *AtFtsZ2* and *AtFtsZ1* were amplified using LY332/LY333 and LY330/LY331, and were inserted into the digested pPICZ A-mCerulean backbone. To obtain constructs expressing MTS, the EcoRI/KpnI digested pPICZ A-eYFP-MTS was used as backbone. The primer sets LY332/LY335, LY330/LY334, and LY34/LY35 were used to amplify corresponding fragments in order to yield pPICZ A-AtFtsZ1_C_-eYFP-MTS, pPICZ A-AtFtsZ2_C_-eYFP-MTS, and pPICZ A-GsFtsZA_FL_-eYFP-MTS.

The construction of the co-expression plasmid pPICZ A-AtFtsZ1_FL_-mCerulean-AtFtsZ2_FL_-eYFP-MTS was described previously (Yoshida et al., 2016). To generate pPICZ A-AtFtsZ1_C_-mCerulean-AtFtsZ2_C_-eYFP-MTS, the *AtFtsZ1_C_-mCerulean* expression cassette was amplified using primers CC229/LY190 and then inserted into the BglII digested pPICZ A-AtFtsZ2_C_-eYFP-MTS backbone. To obtain pPICZ A-GsFtsZA_FL_-mCerulean-GsFtsZB_FL_-eYFP, we first amplified the *GsFtsZB_FL_-eYFP-MTS* expression cassette using primers CC229/LY190 and then inserted into the BglII digested pPICZ A-GsFtsZA_FL_-mCerulean backbone. The obtained plasmid was further digested with BamHI/KpnI to excise the *eYFP-MTS* fragment and thus served as backbone. Finally, we used the primer pairs LY201/LY328 and LY329/LY192 to amplify the *eYFP* expression cassette lacking *MTS*, and inserted the cassette into the BamHI/KpnI digested backbone to yield pPICZ A-GsFtsZA_FL_-mCerulean-GsFtsZB_FL_-eYFP. Similar strategy was adopted to generate pPICZ A-GsFtsZA_FL_-mCerulean-GsFtsZB_C_-eYFP. In brief, the *GsFtsZB_C_-eYFP-MTS* expression cassette was first amplified using primers CC229/LY190 and the obtained fragment was inserted into the BglII digested pPICZ A-GsFtsZA_FL_-mCerulean backbone. The obtained plasmid was further digested with BamHI/KpnI to excise the *eYFP-MTS* fragment and used as backbone. The primer pairs LY410/LY328 and LY329/LY192 were used to amplify the *eYFP* expression cassette lacking *MTS*, and the resulting fragment was inserted into the digested backbone to obtain pPICZ A-GsFtsZA_FL_-mCerulean-GsFtsZB_C_-eYFP.

To generate the chimeric protein expression vector pPICZ A-AtFtsZ2_NT_-Z1_C_-Z2_CT_-mCerulean, primer pairs of LY336/LY337, LY338/LY339 and LY340/LY341 were used to amplify fragments of *AtFtsZ2_NT_*, *AtFtsZ1_C_* and *AtFtsZ2_CT_*, and then Gibson assembled into the digested pPICZ A-mCerulean backbone. Similarly, LY336/LY343, LY344/LY345, LY346/LY347 were used for amplification fragments of *AtFtsZ1_NT_*, *AtFtsZ2_C_* and *AtFtsZ1_CT_*, and then Gibson assembled into the digested pPICZ A-mCerulean backbone to yield pPICZ A-AtZ1_NT_-Z2_C_-Z1_CT_-mCerulean construct.

To obtain the pET11b-Z2NTZ1_C_Z2CT vector, the fragment of *Z2NTZ1_C_Z2CT* was amplified using the primer pair LY253/LY252 and then assembled into the BamHI/NdeI digested pET11b vector. Likewise, the fragment of *Z1NTZ2_C_Z1CT* was amplified using the primer pair LY251/LY254 and then assembled into the BamHI/NdeI digested pET11b vector to yield pET11b-Z1NTZ2_C_Z1CT.

### Cell culture, transformation and induction of protein expression in *Pichia*

*Pichia pastoris (P. pastoris)* X-33 strain was used in this study. The pPICZ A expression vector (Invitrogen), harboring the genes of interest, was transformed into the X-33 strain and integrated into the host genome via homologous recombination. The transformation of *Pichia* was conducted as previously described, with minor modifications (Chen et al., 2019). Briefly, 10 μg of the pPICZ A vector was linearized using enzyme restriction and introduced into the X-33 strain via electroporation. The pPICZ A vector contains a phleomycin resistant gene for selection in both *Escherichia coli* (*E. coli*) and *P. pastoris*. To select positive transformants in *Pichia*, 10 μg mL^-1^ phleomycin (InvioGen) was added to YPDS (1% yeast extract, 2% peptone, 1 M sorbitol, 2% dextrose) plates containing 2% agar.

The pPICZ A vector features an inducible *AOX1* promoter, which facilitates distinct levels of protein expression. During the growth phase, proteins are minimally expressed in BMGY medium (1% yeast extract, 2% peptone, 100 mM potassium phosphate at pH 6.0, 1.34% YNB, 0.00004% biotin, 1% glycerol), where glucose serves as the sole carbon source. In contrast, protein expression is induced upon transfer to BM medium (1% yeast extract, 2% peptone, 100 mM potassium phosphate at pH 6.0, 1.34% YNB, 0.00004% biotin), which lacks a carbon source.

To assess the expression of FtsZ proteins in *P. pastoris*, transformed *Pichia* cells were streaked out onto YPD (1% yeast extract, 2% peptone, 2% dextrose) plates. A single colony was selected, pre-cultured in 2.5 mL of BMGY medium overnight, and then centrifuge at 1,000 *g* for 2 min at room temperature. The pellet was resuspended in 500 μL of BM medium, and the culture was grown continuously in a shaker at 30°C until the indicated time.

### Expression and purification of the recombinant proteins in *E. coli*

To express the recombinant chimeric His-Z2NTZ1_C_Z2CT and His-Z1NTZ2_C_Z1CT proteins, we transformed the corresponding vectors into *E. coli* DE3 Rosetta cells. The transformed bacterial cells were cultured in a shaker at 37°C overnight. The following day, they were subcultured into fresh LB medium and allowed to grow at 37°C until the OD_600_ reached between 0.6 and 0.8. Subsequently, the cultures were subjected to an ice shock for 10 min before the addition of Isopropyl β-D-1-thiogalactopyranoside (IPTG; 0.6 mM) to induce protein expression. After 36–42 hours of induction at 14°C, the cells were harvested by centrifugation and re-suspended in 20 mL of low salt buffer (LSB: 20 mM Tris pH 7.5, 50 mM NaCl, 10% glycerol). The re-suspended cell pellets were then stored at -80°C for further use.

Purification of the chimeric proteins was conducted according to the method previously described (Porter et al., 2021). In brief, the harvested cells were thawed and lysed using 1 mg/ml Lysozyme (Lab Scientific, Highlands, NJ) for 30 min at 4°C. After lysis, the cells were sonicated to further rupture the cells and release the expressed proteins. Following centrifugation to pellet the cell debris, the supernatant containing the soluble protein was loaded onto a Ni-NTA column (Qiagen). The column was then washed with a gradient of imidazole concentrations (from 20 to 50 mM) in LSB (20 mM Tris pH 7.5, 50 mM NaCl, 10% glycerol) to remove unbound proteins. The chimeric proteins were eluted using 300 mM imidazole in LSB. The eluted proteins were dialyzed against LSB to exchange the buffer and remove imidazole. Finally, the purified proteins were aliquoted and stored at -80°C for long-term preservation.

### GTPase measurement

GTPase activity was measured following the procedures from Ingermann and Nunnari, with slightly modifications (Ingerman and Nunnari, 2005). The assay fundamentally measured the depletion of NADPH at 340 nm. The consumption of one NADPH molecule facilitates the regeneration of a GTP from a GDP, thereby reflecting the GTPase activities. All proteins used in the experiments were centrifuged at 80,000 *g* for 30 min at 4°C prior to the assay. In brief, a total volume of 180 µL was prepared, containing the desired protein concentrations in the reaction buffer (1 mM phosphoenolpyruvate, 0.4 mM NADH, and 20 U/mL pyruvate kinase/lactate dehydrogenase, in 50 mM HEPES–KOH, pH 7.5, 5 mM MgSO4, and 100 mM KCl). Finally, 20 µL of GTP (Sigma) was added to initiate the monitoring of absorbance at 340 nm using a SpectraMax M2 microplate reader (Molecular Devices). The GTPase activities were calculated according to the previous study (Porter et al., 2021).

### Microscopy imaging and FRAP analysis

All images were collected at room temperature using a spectral-based FluoView 1000 laser scanning confocal microscope (Olympus) equipped with a UPlanSApo 100× (NA 1.40) oil immersion objective. FV1000 ASW software (Olympus) was used to capture either snapshots or time-lapse images during the FRAP experiment. For proteins fused with eYFP in single expression cells, a 515 nm laser was utilized, while for proteins fused with mCerulean, a 458 nm laser was utilized. FRAP analysis was conducted as previously described (Yoshida et al., 2016; Chen et al., 2019), with slight modifications. Briefly, three images with a 10 s interval were taken as the pre-bleached control, and then a ROI (Region of Interest) of 20 pixels in diameter was selected for photobleaching for 20 ms using the Tornado scanning tool within the FV1000 ASW software. Recovery of the fluorescence signals was monitored at 10 s intervals for a total of 260 s. To correct and normalize the FRAP data, ROIs with the same size were recorded during each experiment: one at a region of the fluorescence signal away from the photobleached spot to account for photobleaching due to continual acquisition, and another at a region of the background signal to account for random noise. Curve fitting was performed with ProFit 7 software. The data were fit to the function of a two-binding-state model (Sprague et al., 2004; Yoshida et al., 2016): *f*(*t*) = (1 - *r*) (1 - C_eq1_ e^-koff1*t^ - C_eq2_ e^-koff2*t^), where *t* is time (s), *C_eq1_* and *C_eq2_* refer to the fractions of bound molecules, *k_off1_* and *k_off2_* refer to dissociation rate constants, and *r* is an additional parameter to account for incomplete recovery (Sprague et al., 2004). The corresponding parameters for each FRAP data were summarized in **Supplementary Tables S1**, **S2**. All image processing was conducted in Fiji.

### Accession numbers

The GenBank accession numbers for the *FtsZ* genes are as follows: cDNAs of *A. thaliana FtsZ2* (AF089738) and *A. thaliana FtsZ1* (AY113896) and *G. sulphuraria FtsZA* (BBAA82099), and *G. sulphuraria FtsZB* (BAA82091).

## Results

### The conserved core regions determine the distinct dynamics of AtFtsZ1 and AtFtsZ2 filaments

**Figure 1:**
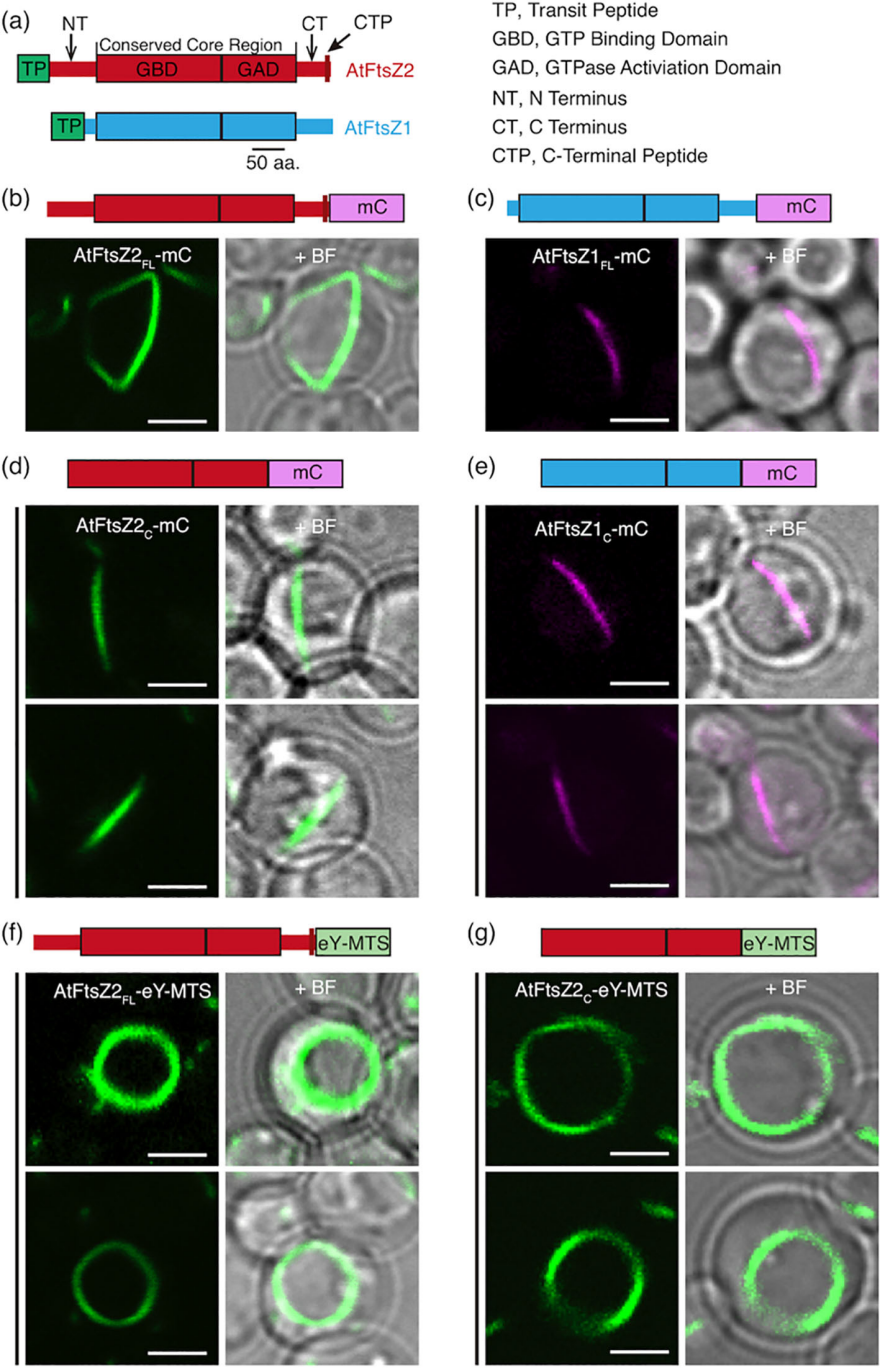
AtFtsZ Filament and Ring Morphologies in *P. pastoris*. **(a)** Structural features of AtFtsZ2 (top) and AtFtsZ1 (bottom). aa, amino acids. **(b, c)** Representative images of full-length *A*. *thaliana* FtsZs, lacking their predicted transit peptides (TPs) (Olson et al., 2010), expressed separately in *P. pastoris*. Specifically, the FtsZs expressed are as follows: FtsZ2-mCerulean (AtFtsZ2_FL_-mC), and FtsZ1-mCerulean (AtFtsZ1_FL_-mC). **(b)** AtFtsZ2_FL_-mC formed deep-curved but unclosed filamentous structures, while **(c)** AtFtsZ1_FL_-mC formed shallow-curved filaments. **(d, e)** Representative images of the core regions of AtFtsZ2-mCerulean (AtFtsZ2_C_-mC) or AtFtsZ1-mCerulean (AtFtsZ1_C_-mC) expressed separately in *P. pastoris*. Only shallow-curved filamentous structures were observed in cells expressing either **(d)** AtFtsZ2_C_-mC or **(e)** AtFtsZ1_C_-mC. **(f, g)** Representative images of FtsZ2_FL_-eYFP or FtsZ2_C_-eYFP fused to a membrane-tethering sequence (MTS) derived from *Escherichia coli* MinD (Szeto et al., 2003) at the C terminus (AtFtsZ2_FL_-eY-MTS), allowing AtFtsZ2_FL_-eY or FtsZ2_C_-eY filaments to associate with the membrane (Yoshida et al., 2016). Circular filamentous structures were observed in cells expressed with either **(f)** AtFtsZ2_FL_-eY-MTS or **(g)** AtFtsZ2_C_-eY-MTS. + BF, merge of fluorescent image with corresponding bright field. Bars are 2 μm.

The conserved core regions of AtFtsZ1 and AtFtsZ2 largely determine the distinct assembly properties of the purified proteins *in vitro* (Porter et al., 2021). We therefore asked whether the core regions govern the unique turnover dynamics of their assembled filaments in living cells. To test this, we fused mCerulean (mC) to the C-termini of full-length AtFtsZ1 (AtFtsZ1_FL_) and AtFtsZ2 (AtFtsZ2 _FL_) lacking the TPs (**Figure 1a**) to create AtFtsZ1_FL_-mC and AtFtsZ2_FL_-mC, as well as to their core regions (AtFtsZ1_C_ and AtFtsZ2_C_) (Porter et al., 2021) to create AtFtsZ1_C_-mC and AtFtsZ2_C_-mC. The fusion proteins were then expressed separately in *P. pastoris*. Throughout this paper, we describe data for AtFtsZ2 before AtFtsZ1 because AtFtsZ2 is a more typical FtsZ as described above (TerBush et al., 2013), and to facilitate comparisons between the Arabidopsis and red algal FtsZ proteins in experiments described below.

In control experiments, AtFtsZ2_FL_-mC and AtFtsZ1_FL_-mC both formed filaments in *P. pastoris* resembling those reported previously (Yoshida et al., 2016). AtFtsZ2_FL_-mC filaments looped around the cell and were generally longer and more jagged in appearance than AtFtsZ1_FL_-mC filaments, which were shaped like shallow arcs (**Figures 1b, c**). AtFtsZ2_C_-mC and AtFtsZ1_C_-mC both formed arc-shaped filaments (**Figures 1d, e**). The latter results validated that the core regions alone are sufficient for assembly of both AtFtsZs in living cells (TerBush et al., 2016).

**Figure 2:**
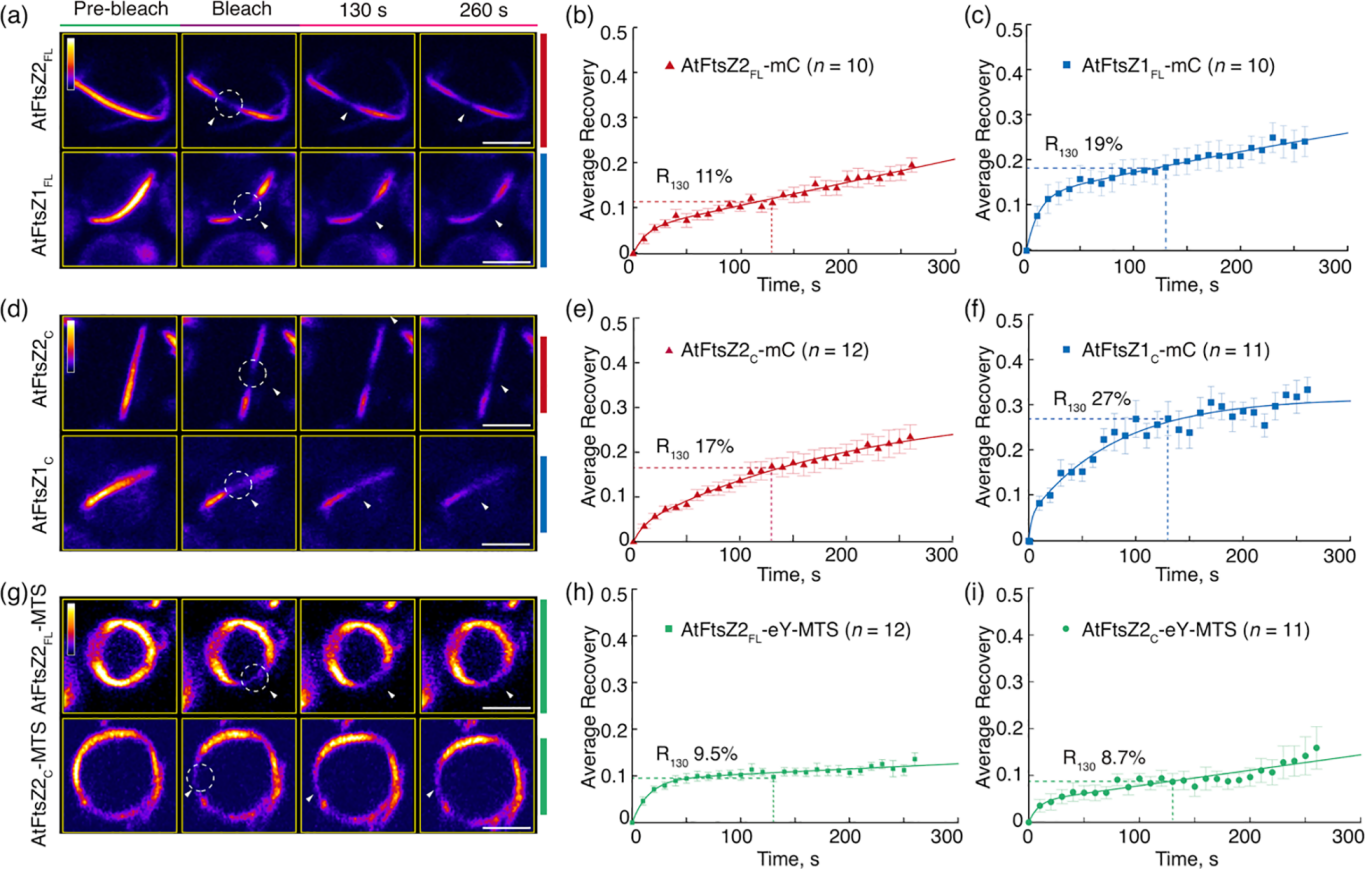
The Conserved Core Regions Determine the Distinct Turnover Dynamics of AtFtsZ1 and AtFtsZ2 Filaments. FRAP (fluorescence recovery after photobleaching) analysis of the AtFtsZ filaments and rings in *Pichia*. FRAP experiments were conducted with either **(a–f)** mCerulean (mC) or **(g–i)** eYFP (eY) signals in *Pichia* cells expressing AtFtsZ proteins separately, as described in **Figure 1**. Recovery of mC or eY fluorescence was recorded for 260 s following photobleaching. A two-binding-state equation was used to perform curve fitting using averaged recovery data (Sprague et al., 2004; Yoshida et al., 2016). R_130_ refers to the percentage of fluorescence recovered at 130 s post-bleaching. **(a, d, g)** Fluorescence images show AtFtsZ filaments and rings prior to photobleaching (Pre-bleach), at the time of photobleaching (Bleach), and at 130 and 260 s after photobleaching. The photobleached regions are indicated by white circles and arrowheads. The fluorescence intensity is indicated by the color scale bar with white the highest and black the lowest. Bars are 2 μm. Curve-fitting graphs derived from FRAP data collected from **(b)** AtFtsZ2_FL_-mC filaments, **(c)** AtFtsZ1_FL_mC filaments, **(e)** AtFtsZ2_C_-mC filaments, **(f)** AtFtsZ1_C_-mC filaments, **(h)** AtFtsZ2_FL_-eY-MTS rings, and **(i)** AtFtsZ2_C_-eY-MTS rings. Dashed lines show the average recovery of fluorescence 130 s after photobleaching (R_130_). Values represent mean ± SE; *n* indicates the number of FRAP cells.

To compare the turnover dynamics of the filaments formed in *P. pastoris*, we performed fluorescence recovery after photobleaching (FRAP) experiments. Recovery of fluorescence into the bleached region was monitored for 260 s and a two-binding-state equation was used to fit the recovery curves (Sprague et al., 2004; Yoshida et al., 2016). Because a half-time of recovery cannot be derived from such curves, we instead used the percent of fluorescence recovered 130 s after photobleaching (R_130_) to allow direct statistical comparisons of dynamics between different FtsZ filaments, and indicated the statistics in **Supplementary Figure S2** (Arabidopsis) and **Supplementary Figure S6** (red alga). The R_130_ values for AtFtsZ2_FL_-mC and AtFtsZ1_FL_-mC were 11% and 19%, respectively (**Figures 2a-c**; **Supplementary Figure S2A**), indicating that AtFtsZ2_FL_ filaments are significantly less dynamic than AtFtsZ1_FL_ filaments, as shown previously in *S. pombe* (TerBush and Osteryoung, 2012). Similarly, AtFtsZ2_C_-mC (R_130_ = 17%) was significantly less dynamic than AtFtsZ1_C_-mC (R_130_ = 27%) (**Figures 2d–f**; **Supplementary Figure S2A**), revealing that the conserved core regions contribute substantially to the differences between AtFtsZ2 and AtFtsZ1 turnover dynamics. R_130_ was significantly lower for AtFtsZ2_FL_-mC (11%) than AtFtsZ2_C_-mC (17%) (**Figures 2b, e**; **Supplementary Figures S2A**, **S3A**), suggesting the N- and/or C-terminal flanking regions may constrain the turnover of FtsZ2 filaments. R_130_ was also lower for AtFtsZ1_FL_-mC (19%) than AtFtsZ1_C_-mC (27%) (**Figures 2c, f**), but the difference was not statistically significant (**Supplementary Figure S2A**). However, the fluorescence recovery curve of AtFtsZ1_FL_-mC exhibited a higher recovery trend than that of AtFtsZ1_C_-mC (**Supplementary Figure S3B**), implying that the N- and/or C-terminal flanking regions may also constrain the turnover of FtsZ1 filaments.

### Membrane tethering of AtFtsZs promotes ring formation

To study constriction of *Escherichia coli* FtsZ *in vitro*, Osawa et al. (2008) fused a membrane-tethering sequence (MTS) to the C-terminus of an FtsZ-eYFP fusion protein, which allowed it to form a membrane-tethered contractile ring inside liposomes. The chloroplast membrane proteins that interact with AtFtsZ2 are lacking in *P. pastoris*, but the same MTS also enabled full-length AtFtsZ2 fused to eYFP (AtFtsZ2_FL_-eY-MTS) to form a contractile ring in *P. pastoris* that was attached to the plasma membrane, whereas AtFtsZ2_FL_ without the MTS was not attached (Yoshida et al., 2016). We used AtFtsZ2_FL_-eY-MTS and constructed AtFtsZ2_C_-eY-MTS to explore the effect of membrane tethering on the assembly and dynamics of the full-length and core AtFtsZ2 proteins. Unlike the proteins lacking the MTS (**Figures 1b, d**), both AtFtsZ2_FL_-eY-MTS and AtFtsZ2_C_-eY-MTS assembled into well-defined closed rings (**Figures 1f, g**). FRAP experiments showed that the R_130_ values for AtFtsZ2_FL_- eY-MTS and AtFtsZ2_C_-eY-MTS were 9.5% and 8.7%, respectively (**Figures 2g–i**; **Supplementary Figure S2B**). Statistical analysis demonstrated that the difference between the dynamics of AtFtsZ2_FL_-eY-MTS and AtFtsZ2_C_-eY-MTS was not significant (**Supplementary Figure S2B**), further indicating that AtFtsZ2 dynamics is dominated by its core region. To further explore the effect of membrane tethering on the assembly and dynamics of AtFtsZ1, we used AtFtsZ1_FL_-eY-MTS (Yoshida et al., 2016) and constructed AtFtsZ1_C_-eY-MTS. Unlike AtFtsZ1_C_-eY-MTS, which was not successfully expressed, AtFtsZ1_FL_-eY-MTS was successfully expressed and assembled into well-defined closed rings (**Supplementary Figure S4A**). The R_130_ value for AtFtsZ1_FL_-eY-MTS was 14% (**Supplementary Figure S4B**). Statistical analysis demonstrated that AtFtsZ1_FL_-eY-MTS rings were more dynamic than AtFtsZ2_FL_-eY-MTS rings (**Supplementary Figure S2B**), suggesting that membrane tethering does not alter the dynamic property difference between AtFtsZ2 and AtFtsZ1. Together, these results suggest that membrane tethering promotes the formation of AtFtsZ2_FL_, AtFtsZ2_C_ and AtFtsZ1_FL_ into rings.

### The conserved core region of AtFtsZ1 enhances AtFtsZ2 dynamics in coassembled filaments

**Figure 3:**
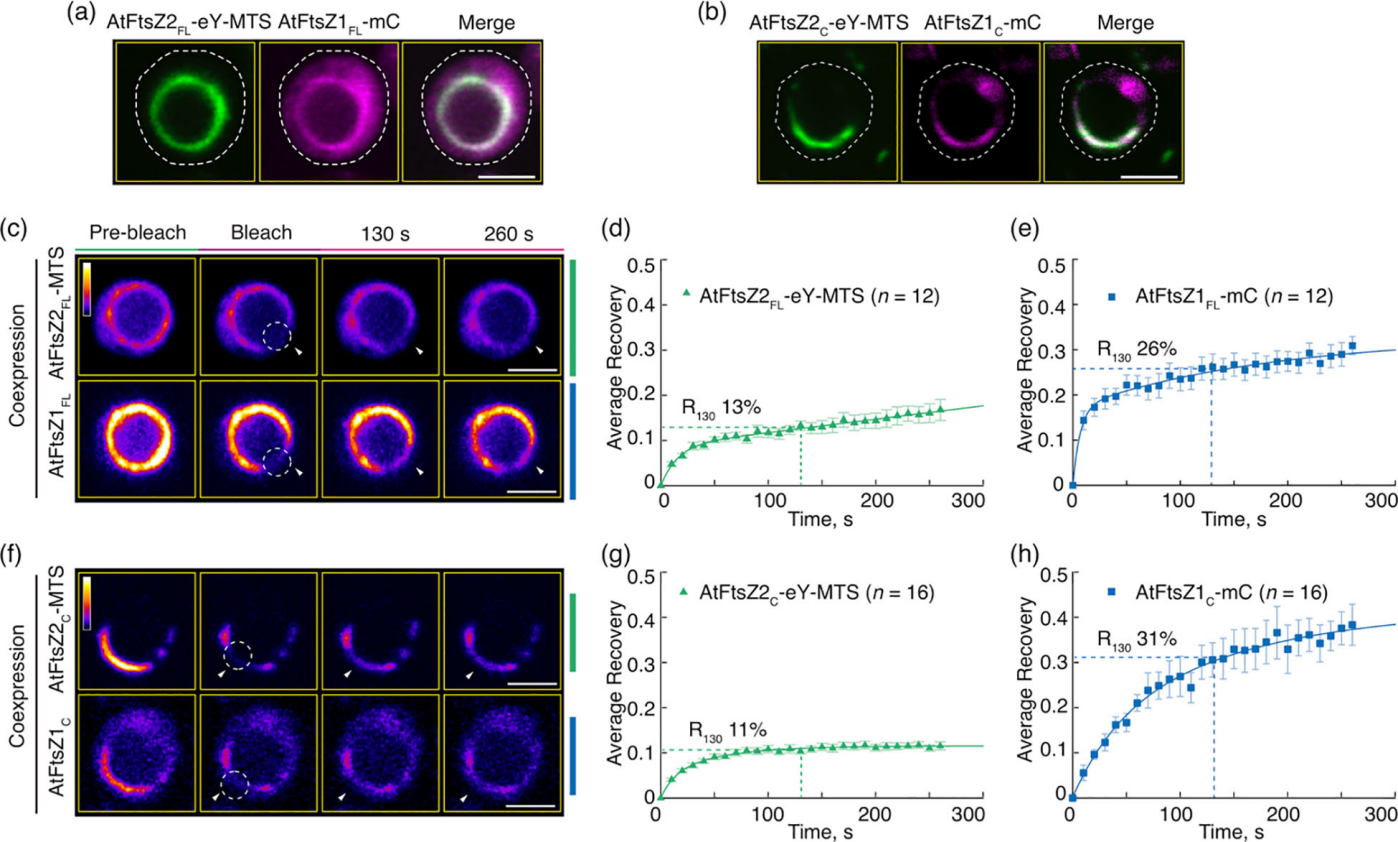
The Conserved Core Regions Are Sufficient for the Dynamic Regulation of Coassembled AtFtsZ1/AtFtsZ2 Filaments. **(a, b)** Coexpression of **(a)** the full-length AtFtsZ1-mCerulean (AtFtsZ1_FL_-mC) and AtFtsZ2_FL_-eYFP-MTS (AtFtsZ2_FL_-eY-MTS), or **(b)** the core regions of AtFtsZ1-mCerulean (AtFtsZ1c-mC) and AtFtsZ2_C_-eYFP-MTS (AtFtsZ2c-eY-MTS) in *Pichia*. White dashed lines indicate the outline of *Pichia* cells. Bars are 2 μm. **(c–h)** FRAP analysis of AtFtsZ rings and filaments when coexpressed in *Pichia*. FRAP experiments were conducted with either eYFP (eY) for AtFtsZ2 or mCerulean (mC) for AtFtsZ1. **(c, f)** Fluorescence images show AtFtsZ rings and filaments prior to photobleaching (Pre-bleach), at the time of photobleaching (Bleach), and at 130 and 260 s after photobleaching. The photobleached regions are indicated by white circles and arrowheads. The fluorescence intensity is indicated by a color scale bar with white the highest and black the lowest. Bars are 2 μm. Curve-fitting graphs derived from the FRAP data collected simultaneously from coexpression of **(d)** AtFtsZ2_FL_-eY-MTS and **(e)** AtFtsZ1_FL_-mC rings, or from coexpression of **(g)** AtFtsZ2_C_-eY-MTS and **(h)** AtFtsZ1_C_-mC filaments. Dashed lines show the average recovery of fluorescence 130 s after photobleaching (R_130_). Values represent mean ± SE; *n* indicates the number of FRAP cells.

AtFtsZ2 is similar to bacterial FtsZs in that its primary function is to establish the structural framework of the chloroplast Z ring (Yoder et al., 2007; Erickson et al., 2010; Chen et al., 2018). In contrast, a critical role for AtFtsZ1 is to enhance Z-ring turnover dynamics by promoting AtFtsZ2 subunit exchange (TerBush and Osteryoung, 2012; Chen et al., 2018; TerBush et al., 2018). To ask whether the core region of AtFtsZ1 is sufficient for this activity, we coexpressed the full-length and core proteins in *P. pastoris* and compared the dynamics of the resulting filaments (**Figures 3a–h**). To mimic the membrane-tethering function of AtFtsZ2 in chloroplasts (Maple et al., 2005; Yoshida et al., 2016), in these experiments we only used AtFtsZ2_FL_-eY-MTS and AtFtsZ2_C_-eY-MTS.

In control experiments, AtFtsZ2_FL_-eY-MTS and AtFtsZ1_FL_-mC coassembled primarily into closed rings (**Figure 3a**) as observed previously (Yoshida et al., 2016). R_130_ for AtFtsZ2_FL_-eY-MTS was significantly higher when coassembled (13%) compared to when assembled separately (9.5%) (**Figures 3c, d**, **2h**; **Supplementary Figure S2B**), which is consistent with prior findings showing that AtFtsZ2 dynamics is accelerated by coassembling with AtFtsZ1. AtFtsZ2_C_-eY-MTS and AtFtsZ1_C_-mC also coassembled, forming either deeply curved arcs wrapped around the cell or closed rings (**Figures 3b, f**). R_130_ for AtFtsZ2_C_-eY-MTS was also significantly higher when coassembled (11%) with AtFtsZ1_C_-mC compared to when assembled separately (8.7%) (**Figures 3g**, **2i**; **Supplementary Figure S2B**). These results demonstrate that the core region of AtFtsZ1 contains the primary information required for enhancing the turnover of AtFtsZ2 subunits from coassembled rings.

### The flanking regions have little influence on the relative dynamics of AtFtsZ2 and AtFtsZ1

**Figure 4:**
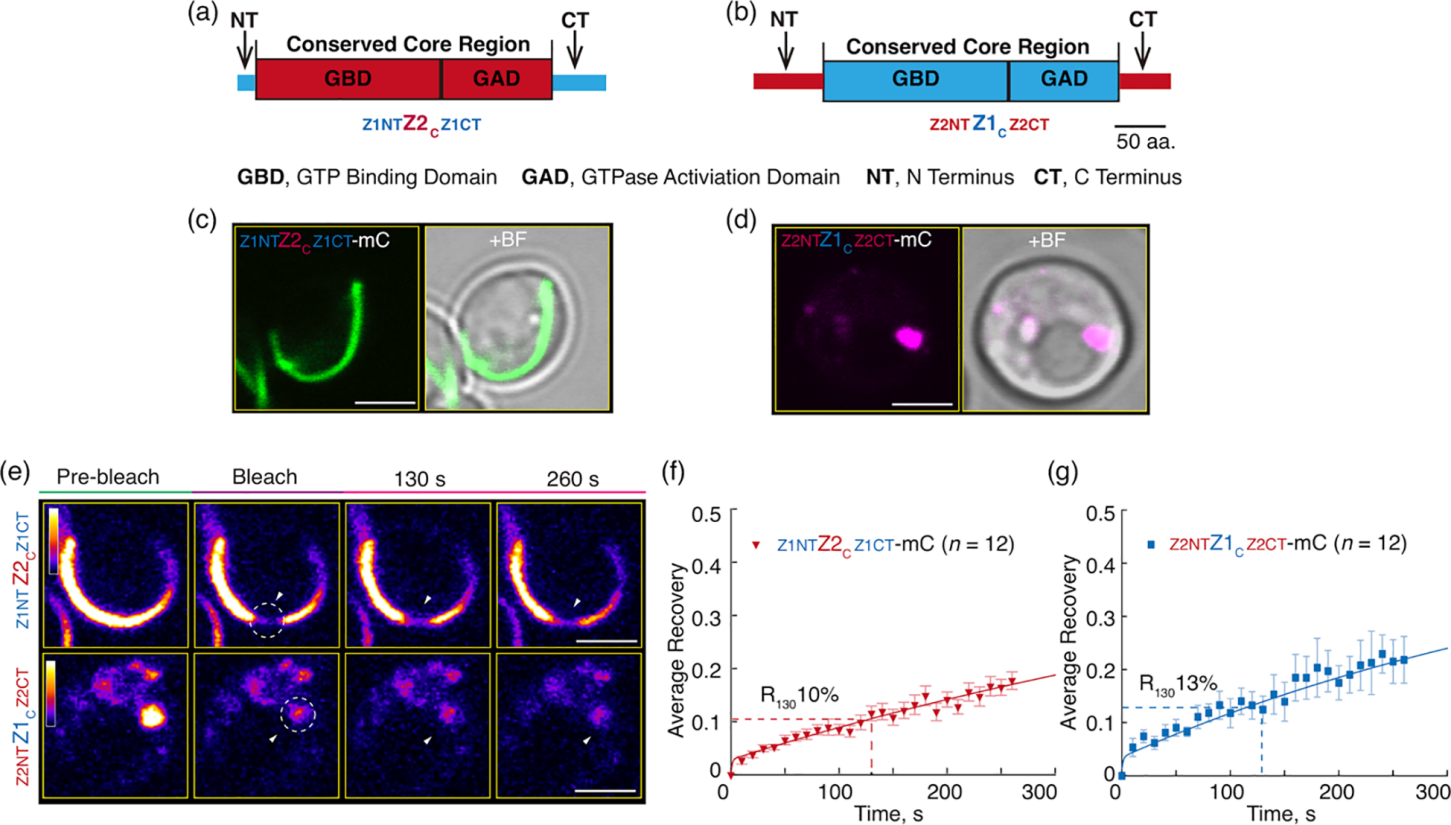
Flanking Regions Have Only Minor Influence on the Relative Dynamics of AtFtsZ1 and AtFtsZ2 Filaments. **(a, b)** Diagrams depicting the structures of the chimeric proteins **(a)** AtFtsZ2_NT_-Z1_C_-Z2_CT_ (Z2NTZ1_C_Z2CT) and **(b)** AtFtsZ1_NT_-Z2_C_-Z1_CT_ (Z1NTZ2_C_Z1CT). aa, amino acids. **(c, d)** Representative images of the chimeras, **(c)** Z2NTZ1_C_Z2CT-mCerulean (Z2NTZ1_C_Z2CT-mC) or **(d)** Z1NTZ2_C_Z1CT-mCerulean (Z1NTZ2_C_Z1CT-mC) expressed in *Pichia*. + BF, merge of fluorescent image with corresponding bright field. **(e–g)** FRAP analysis of the chimeric proteins Z2NTZ1_C_Z2CT-mC and Z1NTZ2_C_Z1CT-mC when expressed individually in *Pichia*. **(e)** Fluorescence images of the chimeras taken prior to photobleaching (Pre-bleach), at the time of photobleaching (Bleach), and at 130 and 260 s after photobleaching. White circles and arrowheads mark the photobleached regions. The fluorescence intensity of mCerulean (mC) is indicated by a color scale bar with white the highest and black the lowest. Bars are 2 μm. Curve-fitting graphs derived from the FRAP data collected from **(f)** Z2NTZ1_C_Z2CT-mC and **(g)** Z1NTZ2_C_Z1CT-mC. Dashed lines show the average recovery of fluorescence 130 s after photobleaching (R_130_). Values represent mean ± SE; *n* indicates the number of FRAP cells. Bars are as indicated.

While our results above show that the core regions dominate the distinct turnover dynamics of AtFtsZ2 and AtFtsZ1, a previous study of truncated AtFtsZs in *S. pombe* suggested the N- and C-terminal regions influence the dynamics of filaments assembled by corresponding AtFtsZs (TerBush et al., 2016). To further explore the contributions of the N-terminal (NT) and C-terminal (CT) flanking regions to the relative difference of the AtFtsZs dynamics, we generated two chimeric proteins, AtFtsZ1_NT_-AtFtsZ2_C_-AtFtsZ1_CT_ (Z1NTZ2_C_Z1CT) and AtFtsZ2_NT_-AtFtsZ1_C_-AtFtsZ2_CT_ (Z2NTZ1_C_Z2CT), in which the flanking regions were swapped (**Figures 4a, b**). This swap strategy was designed to explore whether the flanking regions of AtFtsZ1 would make AtFtsZ2_C_ behaves like AtFtsZ1 (**Figure 4a**), and *vice versa* (**Figure 4b**). To evaluate their functionalities, we expressed them in *E. coli*, purified them, and measured their GTPase activities. The activities of Z1NTZ2_C_Z1CT and Z2NTZ1_C_Z2CT at 25°C were 0.39 ± 0.21 and 0.26 ± 0.10 GTP FtsZ^-1^ min^-1^, respectively, similar to the activities reported for AtFtsZ2 and AtFtsZ1, respectively (Porter et al., 2021), indicating both chimeras are functional GTPases.

The chimeric proteins were then fused to mCerulean and expressed in *P. pastoris*. Z1NTZ2_C_Z1CT-mC formed long arced filaments while Z2NTZ1_C_Z2CT-mC formed condensates (**Figures 4c, d**), indicating that the swap of AtFtsZ2’s flanking regions to AtFtsZ1_C_ appears to prevent the Z2NTZ1_C_Z2CT chimeric protein from assembling into long filaments. In FRAP experiments, Z1NTZ2_C_Z1CT-mC (R_130_ = 10%) was less dynamic than Z2NTZ1_C_Z2CT-mC (R_130_ = 13%) (**Figures 4e–g**; **Supplementary Figure S3C**). However, the difference in R_130_ values was not statistically significant (**Supplementary Figure S2C**), unlike the difference between AtFtsZ2_FL_-mC and AtFtsZ1_FL_-mC (**Supplementary Figure S2A**). However, the dynamics of Z1NTZ2_C_Z1CT-mC and Z2NTZ1_C_Z2CT-mC were similar to those of AtFtsZ2_FL_-mC and AtFtsZ1_FL_-mC, respectively (**Supplementary Figure S2C**), implying the flanking regions of one protein did not alter the overall dynamics of the other. Both chimeric proteins (**Figures 4f, g**) were significantly less dynamic than their corresponding core proteins (**Figures 2e, f**; **Supplementary Figure S2C**), suggesting that the flanking regions of AtFtsZs generally suppress the dynamics of both proteins. Collectively, these results are consistent with the conclusion that the core regions predominate in determining the differences in the relative dynamics between AtFtsZ2 and AtFtsZ1.

### Subunit exchange dynamics of red algal FtsZs are determined by their conserved core regions

**Figure 5:**
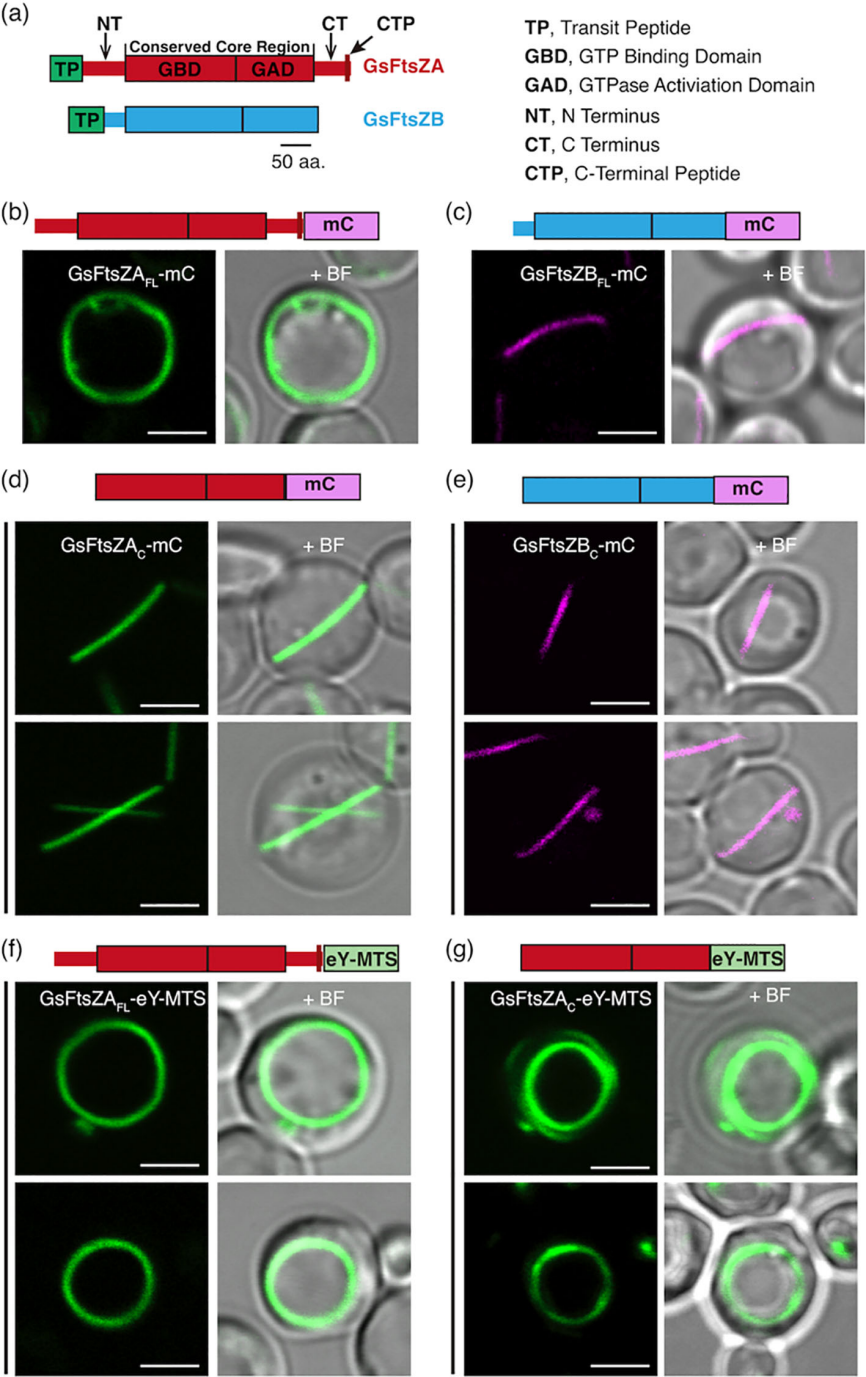
GsFtsZ Filament and Ring Morphologies in *P. pastoris*. **(a)** Structural feature of *G*. *sulphuraria* FtsZA (GsFtsZA; top) and FtsZB (GsFtsZB; bottom). aa, amino acids. **(b, c)** Representative images of full-length *G*. *sulphuraria* FtsZs, lacking their predicted transit peptides (TPs) (Olson et al., 2010), expressed separately in *Pichia*. Specifically, the FtsZs expressed are as follows: GsFtsZA-mCerulean (GsFtsZA_FL_-mC), and GsFtsZB-mCerulean (GsFtsZB_FL_-mC). **(b)** GsFtsZA_FL_-mC formed ring-like structures around the cell boundary, while **(c)** the GsFtsZB_FL_-mC assembled into straight filament. **(d, e)** Representative images of the core regions of GsFtsZA-mCerulean (GsFtsZA_C_-mC) or GsFtsZB-mCerulean (GsFtsZB_C_-mC) expressed separately in *P. pastoris*. Only shallow-curved filamentous structures were observed in cells expressing either **(d)** GsFtsZA_C_-mC or **(e)** GsFtsZB_C_-mC. **(f, g)** Representative images of GsFtsZA_FL_-eYFP or GsFtsZA_C_-eYFP fused to a membrane-tethering sequence (MTS) derived from *Escherichia coli* MinD (Szeto et al., 2003) at the C terminus. Fusion of MTS to GsFtsZA_C_-eY (GsFtsZA_C_-eY-MTS) led to the formation of ring-like structures around the cell boundary. + BF, merge of fluorescent image with corresponding bright field. Bars are 2 μm.

**Figure 6:**
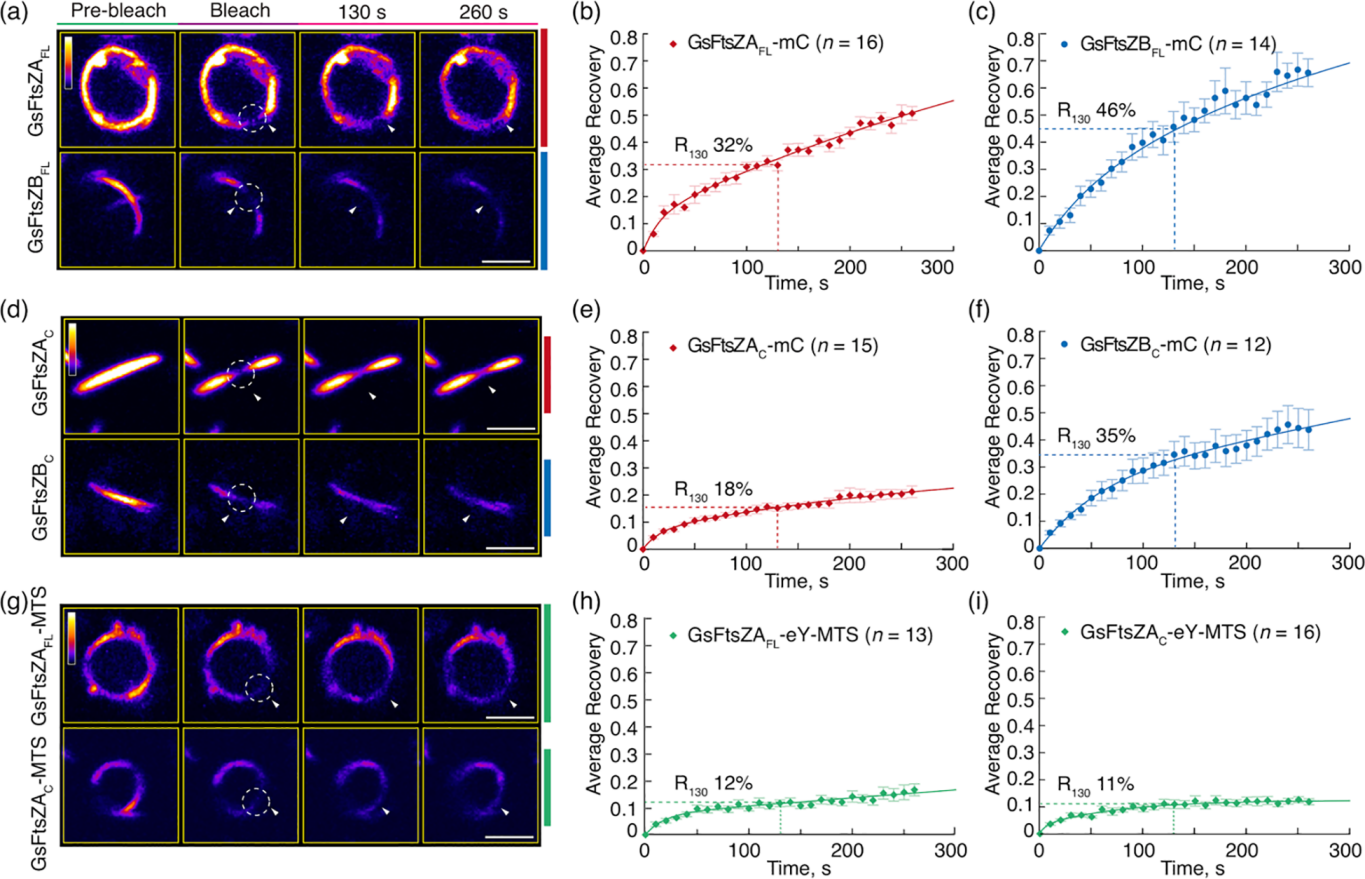
The Core Regions Determine the Distinct Dynamics of Red Algal FtsZA and FtsZB Filaments in *Galdieria sulpuraria*. FRAP analysis of the GsFtsZ rings and filaments in *Pichia*. FRAP experiments were conducted with either **(a–f)** mCerulean (mC) or **(g–i)** eYFP (eY) signals in *Pichia* cells expressing GsFtsZ proteins separately, as described in **Figure 5**. Recovery of mC or eY fluorescence was recorded for 260 s following photobleaching. A two-binding-state equation was used to perform curve fitting using averaged recovery data (Sprague et al., 2004; Yoshida et al., 2016). **(a, d, g)** Fluorescence images show GsFtsZ rings and filaments prior to photobleaching (Pre-bleach), at the time of photobleaching (Bleach), and at 130 and 260 s after photobleaching. The photobleached regions are indicated by white circles and arrowheads. The fluorescence intensity is indicated by a color scale bar with white the highest and black the lowest. Bars are 2 μm. Curve-fitting graphs derived from FRAP data collected from **(b)** GsFtsZA_FL_-mC rings, **(c)** GsFtsZB_FL_mC filaments, **(e)** GsFtsZA_C_-mC filaments, **(f)** GsFtsZB_C_-mC filaments, **(H)** GsFtsZA_FL_-eY-MTS rings, and **(i)** GsFtsZA_C_-eY-MTS rings. Dashed lines show the average recovery of fluorescence 130 s after photobleaching (R_130_). Values represent mean ± SE; *n* indicates the number of FRAP cells.

The dominant role of the core regions in controlling AtFtsZ filament turnover dynamics led us to ask if this also applies to other chloroplast FtsZ pairs. To address this, we tagged full-length *Galdieria sulphuraria* (Gs) FtsZA (GsFtsZA_FL_) and GsFtsZB (GsFtsZB_FL_) and their corresponding core regions (GsFtsZA_C_ and GsFtsZB_C_) with mCerulean (**Figure 5a**). These constructs did not include the TP of corresponding GsFtsZs, and each construct was expressed individually in *P. pastoris*. GsFtsZA_FL_-mC assembled into closed ring-like structures while GsFtsZB_FL_-mC formed straight or arc-shaped filaments (**Figures 5b, c**). In FRAP experiments, the fluorescence recovery curve of GsFtsZA_FL_-mC exhibited a slower recovery trend compared to that of GsFtsZB_FL_-mC (**Figures 6a–c**; **Supplementary Figure S5A**). The R_130_ values for GsFtsZA_FL_-mC and GsFtsZB_FL_-mC were 32% and 46%, respectively. The difference between these values was statistically significant (**Supplementary Figure S6A**), indicating that GsFtsZA_FL_-mC filaments are significantly less dynamic than GsFtsZB_FL_-mC filaments. While the core proteins of GsFtsZA_C_-mC and GsFtsZB_C_-mC both assembled into shallow arcs (**Figures 5d, e**), R_130_ values for GsFtsZA_C_-mC filaments (18%) were significantly lower than for GsFtsZB_C_-mC filaments (35%) (**Figures 6d–f**; **Supplementary Figure S6A**), indicating that the GsFtsZ core proteins behaved similarly to the full-length proteins. Combined with the data from AtFtsZ, these findings suggest that the core regions of FtsZs dominate their unique dynamics in living cells. However, both GsFtsZ core proteins were less dynamic than their corresponding full-length proteins (**Supplementary Figures S5B, C**), although statistically the difference was only significant for GsFtsZA_FL_-mC (R_130_ = 32%) *vs.* GsFtsZA_C_-mC (R_130_ = 18%) (**Supplementary Figure S6A**). These differences are opposite those observed between the AtFtsZ core and full-length proteins, and suggest that the flanking regions of the GsFtsZ proteins, particularly for GsFtsZA, influence their absolute dynamics.

To investigate how membrane association affects the morphology and dynamics of the GsFtsZA full-length and core proteins, we generated GsFtsZA_FL_-eY-MTS and GsFtsZA_C_-eY-MTS. Both GsFtsZA_FL_-eY-MTS and GsFtsZA_C_-eY-MTS assembled into well-defined rings (**Figures 5f, g**), indicating that MTS fusions facilitate ring formation. R_130_ values for GsFtsZA_FL_-eY-MTS (12%) and GsFtsZA_C_-eY-MTS (11%) were both reduced compared to those of the equivalent proteins lacking the MTS (**Figures 6b, e, g–i**), in particular for GsFtsZA_FL_-mC (32%) though the fluorescent tags were not identical. Together with our AtFtsZ2 results, these data suggest that membrane tethering promotes the Z-ring assembly and likely constrains filament dynamics as well. However, the dynamics of GstFtsZA_FL_-eY-MTS (R_130_ = 12%) and GsFtsZA_C_-eY-MTS (R_130_ = 11%) were very similar (**Figures 6h, i**; **Supplementary Figure S6A**), further supporting that the core regions determine the dynamics of GsFtsZA, as observed for AtFtsZ2 (**Figures 2h, i**; **Supplementary Figure S2B**).

### The conserved core region of GsFtsZB enhances the dynamics of the full-length GsFtsZA in coassembled rings

**Figure 7:**
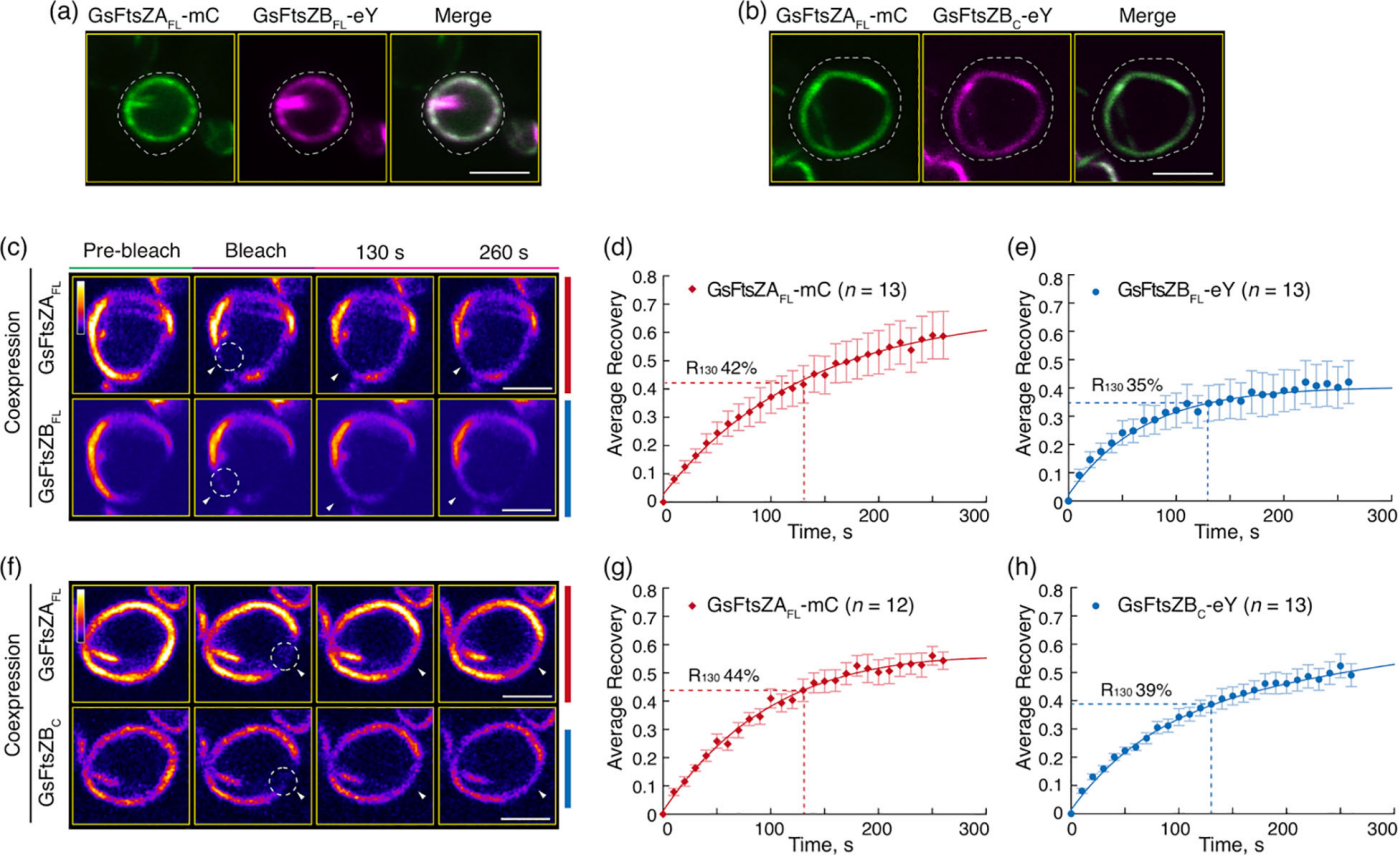
The Core Region of FtsZB Promote the Overall Dynamics of Coassembled Filaments with FtsZA. **(a, b)** Coexpression of **(a)** the full-length GsFtsZA-mCerulean (GsFtsZA_FL_-mC) and GsFtsZB-eYFP (GsFtsZB_FL_-eY), or **(b)** GsFtsZA_FL_-mC and the core region of GsFtsZB-eYFP (GsFtsZB_C_-eY) in *Pichia*. White dashed lines indicate the outline of *Pichia* cells. Bars are 2 μm. **(c–h)** FRAP analysis of GsFtsZ rings when coexpressed in *Pichia*. FRAP experiment was conducted with either mCerulean (mC) for GsFtsZA or eYFP (eY) for GsFtsZB. **(c, f)** Fluorescence images show GsFtsZ rings prior to photobleaching (Pre-bleach), at the time of photobleaching (Bleach), and at 130 and 260 s after photobleaching. The photobleached regions are indicated by white circles and arrowheads. The fluorescence intensity is indicated by a color scale bar with white the highest and black the lowest. Bars are 2 μm. Curve-fitting graphs derived from the FRAP data collected simultaneously from coexpression of **(d)** GsFtsZA_FL_-mC and **(e)** GsFtsZB_FL_-eY, or from coexpression of **(g)** GsFtsZA_FL_-mC and **(h)** GsFtsZB_C_-eY rings. Dashed lines show the average recovery of fluorescence 130 s after photobleaching (R_130_). Values represent mean ± SE; *n* indicates the number of FRAP cells.

It has been proposed that FtsZB in red algae functions similarly to FtsZ1 in green lineage. In line with this, GsFtsZB has been reported to promote exchange of GsFtsZA subunits from coassembled filaments in *S. pombe* (TerBush et al., 2018). To test whether the core region of GsFtsZB is sufficient to enhance the dynamics of red algal chloroplast Z ring, we coexpressed the full-length of GsFtsZA with the full-length or core protein of GsFtsZB in *P. pastoris* and performed FRAP analysis on the reconstituted GsFtsZ rings (**Figures 7a–h**). Given that GsFtsZA_FL_ forms ring-like structures in the absence of MTS (**Figure 6a**), we only used GsFtsZA_FL_-mC in the coexpression experiments. In contrast to singular expression, both GsFtsZB_FL_-eY and GsFtsZB_C_-eY coassembled primarily into ring-like structures when coexpressed with GsFtsZA_FL_-mC (**Figures 7a, b**), further supporting it is GsFtsZA that dominates the morphology of red algal chloroplast Z ring, as AtFtsZ2 does in green lineage.

FRAP analyses showed that R_130_ for GsFtsZA_FL_-mC was significantly increased when coassembled (42%) with GsFtsZB_FL_-eY compared to when assembled separately (32%) (**Figures 7c, d**, **6b**; **Supplementary Figure S6B**), demonstrating that GsFtsZB can enhance the turnover dynamic of GsFtsZA in the reconstituted red algal chloroplast Z ring, as observed for AtFtsZ1 in green lineage. Likewise, R_130_ for GsFtsZA_FL_-mC was significantly increased when coassembled (44%) with GsFtsZB_C_-eY compared to when assembled separately (32%) (**Figures 7f, g**, **6b**; **Supplementary Figure S6B**), indicating an enhancement of GsFtsZA_FL_-mC dynamics by GsFtsZB_C_-eY in the coassembled rings. These findings demonstrate that the core region of GsFtsZB is sufficient to promote the turnover dynamic of GsFtsZA submits from their coassembled rings.

## Discussion

In this study, we have taken advantage of FRAP technology to investigate the dynamic turnover of plant and red algal chloroplast FtsZ proteins in a yeast system. While it has been hypothesized that the less conserved flanking regions of FtsZs might be responsible for the functional differences between the duplicated FtsZ pairs in chloroplasts (TerBush et al., 2016), our findings directly demonstrate that the conserved core regions are the primary determinants of the dynamic behaviors exhibited by FtsZ filaments or rings when reconstituted in yeast.

Previous studies have shown that AtFtsZ2 and AtFtsZ1 coassemble and are both required for chloroplast division (Osteryoung et al., 1998; Olson et al., 2010; TerBush and Osteryoung, 2012). AtFtsZ2 likely determines the morphology and stability of chloroplast FtsZ protofilaments and rings, whereas AtFtsZ1 functions to enhance their turnover dynamics (TerBush and Osteryoung, 2012; Yoshida et al., 2016; TerBush et al., 2018). Here we have shown that AtFtsZ2_C_, similar to AtFtsZ2_FL_, can form ring-like structures when anchored to the cell membrane via an MTS (**Figure 1g**). Moreover, AtFtsZ2_C_ exhibited slower dynamics than AtFtsZ1_C_, as revealed by a direct comparison of the R_130_ values when they were assembled separately with the identical fluorescent tags (**Figures 2e, f**; **Supplementary Figure S2A**). We also demonstrated that AtFtsZ1_C_, like AtFtsZ1_FL_ (TerBush and Osteryoung, 2012), accelerates the turnover dynamics of AtFtsZ2_C_ in coassembled filaments and rings (**Figures 2i**, **3g, h**; **Supplementary Figure S2B**). Consequently, our results indicate that the core regions of AtFtsZ2 and AtFtsZ1 are primarily responsible for their distinct functions. This property appears to extend to the red algal FtsZs, as filaments assembled from the core regions of GsFtsZA and GsFtsZB exhibit behaviors analogous to their full-length counterparts (**Figures 6b, c, e, f**). Recent *in vitro* study has demonstrated that the assembly dynamics of the two FtsZ proteins from Arabidopsis is also governed by their core regions (Porter et al., 2021). These findings are consistent with our current studies and underscore the critical role of the FtsZ core regions in the dynamic behavior of the chloroplast Z ring.

The conserved core regions of FtsZ proteins consist of GTP binding and GTPase activating domains (**Figure 1a**). The GTPase activating domain is situated within the C-terminus of the FtsZ_C_ protein. It has been proposed that the GTPase active site forms at the longitudinal interface between two FtsZ subunits (Scheffers et al., 2002). GTP hydrolysis at this active site weakens the interface, promoting the disassociation of GDP-bound subunits from the protofilaments, which ultimately leads to the turnover dynamics of the FtsZ protofilaments and rings. A recent biochemical study showed that the GTPase activities of AtFtsZ2_C_ and AtFtsZ1_C_ are distinct, with AtFtsZ2_C_ exhibiting lower GTPase activity than AtFtsZ1_C_ (Porter et al., 2021). Consistent with this, we observed that the dynamics of AtFtsZ2_C_ filaments were significantly slower than those of AtFtsZ1_C_ (**Figures 2e, f**; **Supplementary Figure S2A**). Thus, we suggest that the GTPase activating domain within the conserved core regions may play a significant role in determining the turnover dynamics of FtsZ protofilaments. To begin testing this hypothesis, we compared the turnover dynamics of filaments assembled from two chimeric proteins constructed previously to examine AtFtsZ1/AtFtsZ2 heteropolymerization (Yoshida et al., 2016): Z2-Z1 chimera, containing the GTPase activating domain of AtFtsZ1 (**Supplementary Figure S7A**) and Z1-Z2 chimera, containing the GTPase activating domain of AtFtsZ2 (**Supplementary Figure S7B**). Our data indicated that Z2-Z1 chimera (R_130_ = 28%) filaments were more dynamic than Z1-Z2 chimera (R_130_ = 8%) (**Supplementary Figures S7C, D**), suggesting that the GTPase activating domain contributes significantly to the overall turnover dynamics of the chimeric AtFtsZs. We note that because these chimeric proteins retain the C-terminal flanking regions associated with their respective GTPase activating domains (**Supplementary Figures S7A, B**), we cannot rule out the possibility that these regions also influence the dynamics of the chimeric AtFtsZ filaments. However, as shown in **Figures 4f, g** and **Supplementary Figure S2C**, the dynamics of Z1NTZ2_C_Z1CT and Z2NTZ1_C_Z2CT filaments did not differ significantly, suggesting that the C-terminal flanking region of AtFtsZ1 is unlikely to accelerate the dynamics of the chimeric Z2-Z1 filaments. Despite this, the GTPase activating domain within AtFtsZ1_C_ is likely not the sole key factor determining the dynamics of AtFtsZ1 filaments, since mutation of a key amino acid for GTPase activity (D275A) did not completely abolish the dynamics of AtFtsZ1 filaments (TerBush and Osteryoung, 2012; TerBush et al., 2018). This is unlike the effects of a similar mutation in AtFtsZ2 (D332A) (TerBush and Osteryoung, 2012; TerBush et al., 2018). Nevertheless, the GTPase activating domain within AtFtsZs appears to be crucial in regulating the turnover dynamics of AtFtsZ filaments, although the underlying mechanisms remain to be elucidated.

Although the dynamics of the AtFtsZ filaments is primarily governed by their core regions, the flanking regions also appear to have an influence (TerBush et al., 2016). The dynamics of filaments assembled from either chimeric Z1NTZ2_C_Z1CT or Z2NTZ1_C_Z2CT proteins (**Figures 4f, g**) were significantly reduced compared to their corresponding core proteins (**Figures 2e, f**; **Supplementary Figure S2C**), implying that the flanking regions suppress the dynamics of both AtFtsZs. AtFtsZ1_FL_ and AtFtsZ1_C_ exhibited similar dynamics (**Figures 2c, f**; **Supplementary Figure S2A**), whereas the Z2NTZ1_C_Z2CT dynamics were significantly reduced compared to AtFtsZ1_C_ dynamics (**Figures 4g**, **2f**; **Supplementary Figure S2C**), suggesting that the AtFtsZ2 flanking regions impose a stronger effect on restricting turnover dynamics than those from AtFtsZ1. Consistent with this speculation AtFtsZ2_FL_ dynamics were significantly lower than AtFtsZ2_C_ dynamics (**Figures 2b, e**; **Supplementary Figure S2A**). These dynamic differences may be associated with previous findings that the bundling of AtFtsZ2_C_ was reduced compared to AtFtsZ2_FL_, suggesting a role for the flanking regions in protofilament bundling (Porter et al., 2021). Bundling of the FtsZ protofilaments leads to their stabilization and a consequent reduction in dynamics (Shaik et al., 2018). The difference in bundling between full-length and core AtFtsZ2 proteins may be partly due to the absence of positively charged residues at the extreme C-terminus in AtFtsZ2_C_, which are known to promote bundling (Buske and Levin, 2013; TerBush et al., 2016). These residues are not present in the C-terminal flanking region of AtFtsZ1. Such difference may contribute to the distinct dynamics of the filaments assembled from the two AtFtsZs, although the core regions remain the dominant determinants of their dynamic properties.

In general, the morphology and dynamic behavior of red algal GsFtsZs were similar to those of their *A. thaliana* counterparts. However, they exhibited some unique properties. Unlike AtFtsZ2_FL_ (**Figure 1b**), GsFtsZA_FL_ could assemble into a ring-like structure without the need for membrane attachment via an MTS (**Figure 5b**). Moreover, GsFtsZA_FL_ was significantly more dynamic than GsFtsZA_C_ filaments (**Figures 6b, e**; **Supplementary Figure S6A**), suggesting that the flanking regions of GsFtsZA contribute positively to the turnover dynamics of the filaments. These findings contrast with the data from AtFtsZ2, where the flanking regions were shown to suppress the overall dynamics of the filaments (**Figures 2b, e**; **Supplementary Figure S2A**). Interestingly, a prior study indicated that the N-terminal truncated GsFtsZA (GsFtsZA_ΔNT_) displayed relatively slow filament dynamics in *S. pombe* (TerBush et al., 2018). Thus, we propose that the N-terminal flanking region of GsFtsZA plays a role in accelerating the dynamics of GsFtsZA filaments. Nevertheless, the core regions still determine the distinct dynamics of the GsFtsZs filaments. Combined with the results from AtFtsZs, our data provide evidence supporting the proposal that the emergence of a second FtsZ protein is a conserved mechanism across chloroplasts from both green and red lineage, acting to enhance the dynamics of the structurally determinant FtsZ subunits. Furthermore, our results highlight that the core regions are the principal determinants of the unique dynamic characteristics of FtsZs in both green and red lineage.

## Data Availability

The original contributions presented in the study are included in the article/**Supplementary Material**. Further inquiries can be directed to the corresponding authors.
