# Supplementary material for: Chloroplast Z-ring dynamics is governed by conserved core regions of evolutionarily divergent FtsZs
Source: Front Plant Sci. 2025 Jul 30;16:1622675. doi: 10.3389/fpls.2025.1622675 (PMC12343686; doi:10.3389/fpls.2025.1622675)
Supplement: Supplementary file 1 [file DataSheet1.docx]

**Figure S1**

**Figure S1.** **Sequences of the FtsZ Globular Regions Are Conserved across Species in Evolution.**

Alignment of FtsZs from cyanobacterial *Synechococcus elongatus 7942* (FtsZ, AAC26227.1), red algal *Galdieria* *sulphuraria* (FtsZA, BAA82090.1; FtsZB, BAA82091.1), green algal *Ostreococcus tauri* (FtsZ2, XP_003080256.1; FtsZ1, XP_003080788.1), monocot *Oryza sativa* *japonica* (FtsZ2, EEE59586.1; FtsZ1, EEE61856.1), and dicot *Arabidopsis thaliana* (FtsZ2, NP_565839.1; FtsZ1, NP_200339.1) was performed using the online alignment software T-coffee under the condition of homology extension (PSI-Coffee). The cyano and orange underlines highlight the sequences of the GTP binding domain and the GTP activating domain, respectively, which are the most conserved regions across different FtsZs. The C-terminal peptides (CTPs), which have been demonstrated or presumed to be critical for the membrane tethering of the Z ring, are identified in both FtsZ2 from the green algal lineage and FtsZA from the red algal lineage, as well as in the ancestral cyanobacterial FtsZ.

**Figure S2**

**Figure S2. R_130_ Values from FRAP Assays on Filaments and Rings Assembled from AtFtsZ Fusion Proteins Expressed Singularly or in Pairs in *P. pastoris*.**

**(a–c)** The values of the recovery of fluorescence at 130 s after photobleaching (R_130_) were statistically compared between the indicated groups. *P* values were determined using the Student’s t-test and are shown correspondingly. The R_130_ data for AtFtsZ2_FL_-mC, AtFtsZ1_FL_-mC, AtFtsZ2_C_-mC, and AtFtsZ1_C_-mC were identical in **(a)** and **(c)**. mC, mCerulean; eY-MTS, eYFP-MTS; NS, not significant.

**
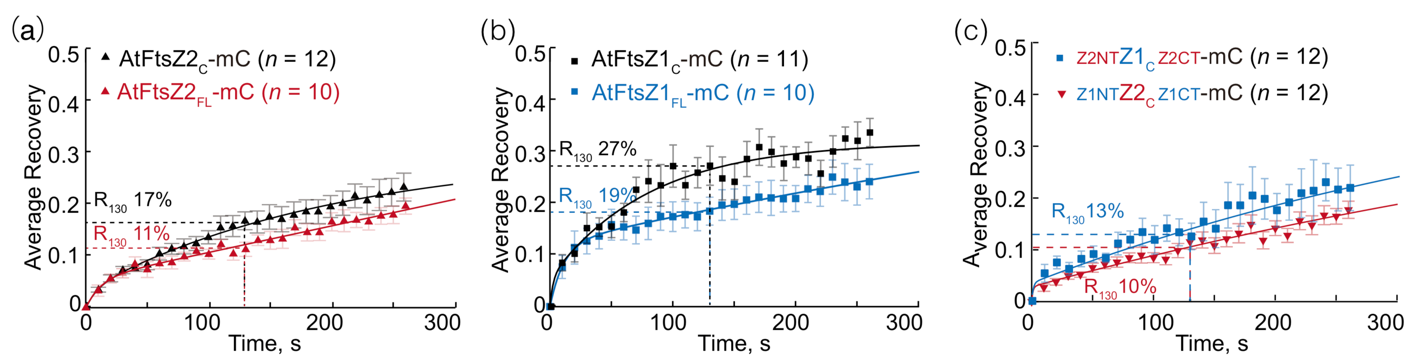
Figure S3**

**Figure S3. FRAP Analysis of AtFtsZ Fusion Proteins Individually Expressed in *P. pastoris*.**

Curve-fitting graphs derived from the FRAP data collected from **(a)** AtFtsZ2_FL_ or AtFtsZ2_C_, **(b)** AtFtsZ1_FL_ or AtFtsZ1_C_, and **(c)** Z1NTZ2_C_Z1CT or Z2NTZ1_C_Z2CT fusion proteins. mC, mCerulean. Dash lines indicate the average fluorescence recovery 130 s after photobleaching (R_130_). Values represent mean ± SE; *n* indicates the number of FRAP cells.

**
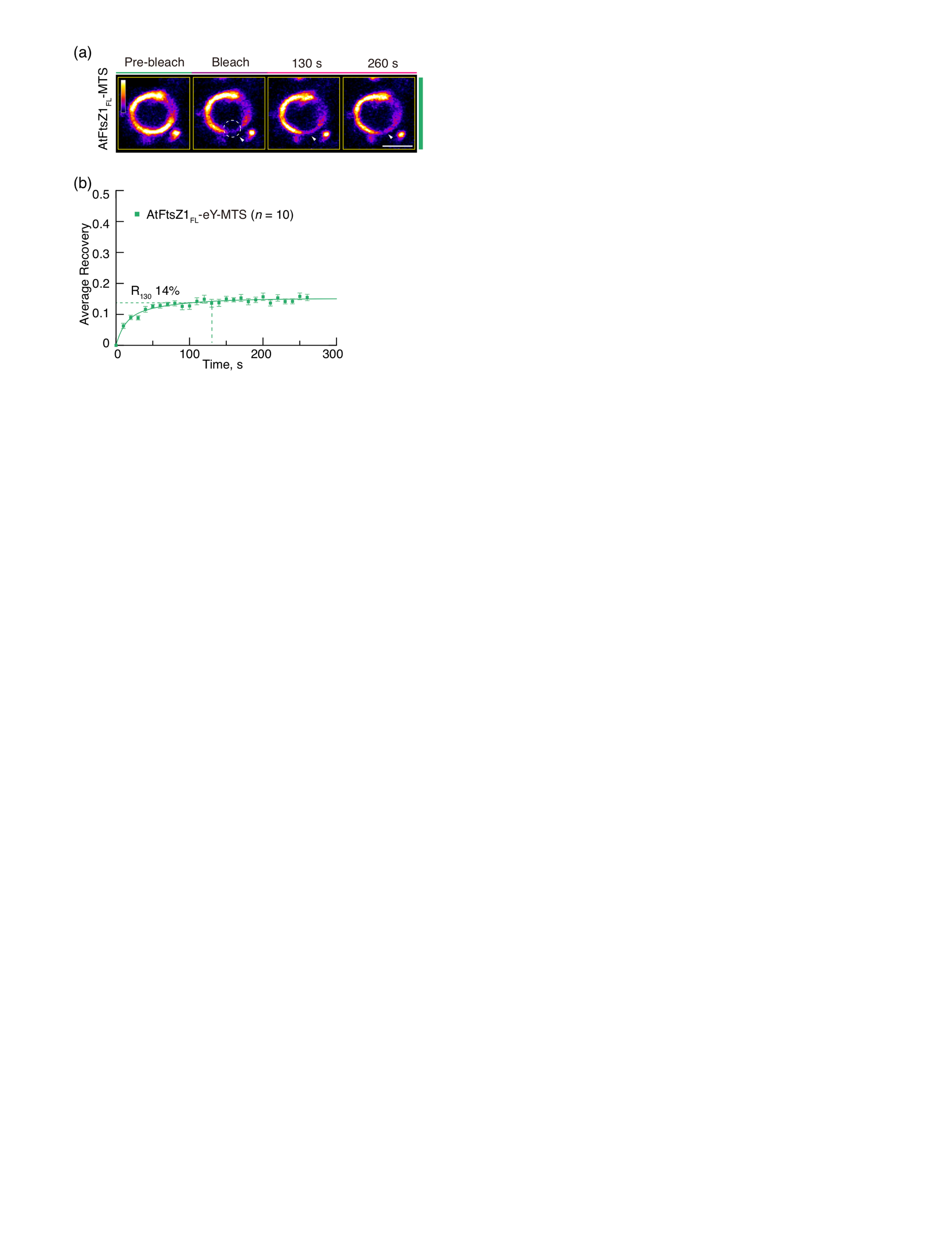
Figure S4**

**Figure S4. Membrane-Tethering of AtFtsZ1 Promotes Ring Formation.**

**(a)** Fluorescence images show AtFtsZ rings prior to photobleaching (Pre-bleach), at the time of photobleaching (Bleach), and at 130 and 260 s after photobleaching. The photobleached regions are indicated by white circles and arrowheads. The fluorescence intensity is indicated by a color scale bar with white the highest and black the lowest. Bars = 2 μm. **(b)** Curve-fitting graphs derived from the FRAP data collected from the AtFtsZ1-eY-MTS rings. Dash lines show the average recovery of fluorescence 130 s after photobleaching (R_130_). Values represent mean ± SE; *n* indicates the number of FRAP cells.

**
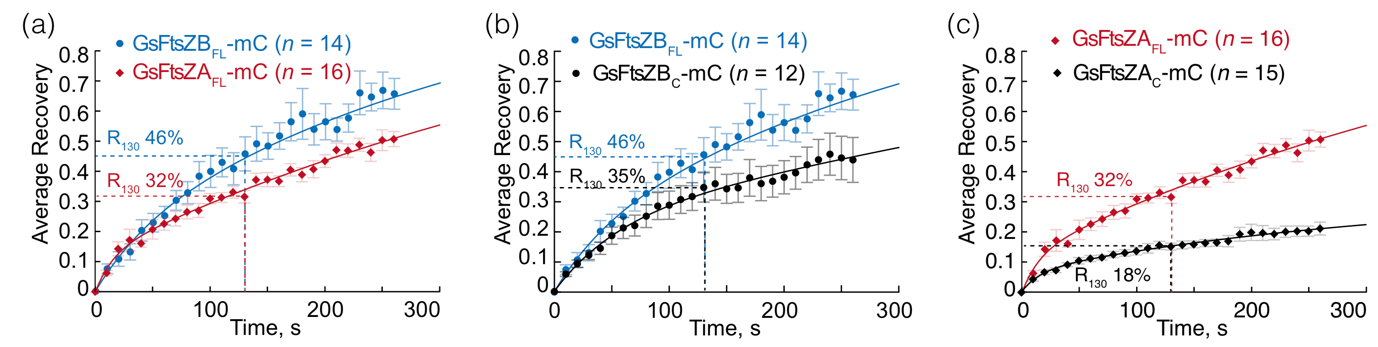
Figure S5**

**Figure S5. FRAP Analysis of GsFtsZ Fusion Proteins Individually Expressed in *P. pastoris*.**

Curve-fitting graphs derived from the FRAP data collected from **(a)** GsFtsZB_FL_ or GsFtsZA_FL_, **(b)** GsFtsZB_FL_ or GsFtsZB_C_, and **(c)** GsFtsZA_FL_ or GsFtsZA_C_ fusion proteins. mC, mCerulean. Dash lines indicate the average fluorescence recovery 130 s after photobleaching (R_130_). Values represent mean ± SE; *n* indicates the number of FRAP cells.

**Figure S6**

**Figure S6. R_130_ Value from FRAP Assays on Rings and Filaments Assembled from GsFtsZ Fusion Proteins Expressed Singularly or in Pairs in *P. pastoris*.**

**(a, b)** The values of the recovery of fluorescence 130 s after photobleaching (R_130_) were statistically compared between the indicated groups. *P* values were determined using the Student’s t-test and are shown accordingly. The R_130_ data for GsFtsZA_FL_-mC was identical for single expression in **(a)** and **(b)**. mC, mCerulean; eY-MTS, eYFP-MTS; exp, expression; NS, not significant.

**Figure S7**

**Figure S7. FRAP Assays of the AtFtsZ2Z1 or AtFtsZ1Z2 Chimeric Proteins Expressed Singularly in *P. pastoris*.**

**(a, b)** Diagram of the **(a)** AtFtsZ2Z1 and **(b)** AtFtsZ1Z2 chimeric proteins. **(c, d)** FRAP assays of the AtFtsZ chimeric proteins when expressed singularly in *P. pastoris*. Curve-fitting graphs derived from FRAP data collected from the **(c)** AtFtsZ2Z1-mCerulean and **(d)** AtFtsZ1Z2-eYFP chimeric proteins. As indicated by the R_130_ values, the turnover dynamics of the AtFtsZ2Z1 chimera was greater than that of the AtFtsZ1Z2 chimera. Values represent mean ± SE; *n* indicates the number of FRAP cells; dashed lines in the graphs indicate R_130_.
