# Supplementary material for: Chloroplast Z-ring dynamics is governed by conserved core regions of evolutionarily divergent FtsZs
Source: Front Plant Sci. 2025 Jul 30;16:1622675. doi: 10.3389/fpls.2025.1622675 (PMC12343686; doi:10.3389/fpls.2025.1622675)
Supplement: Supplementary file 2 [file Table1.docx]

Table S1. FRAP Parameters in Single-Expression Strains.

| Proteins | | *r* | *C_eq1_* | *C_eq2_* | *k_off1_* | *k_off2_* | R_130_ |
| --- | --- | --- | --- | --- | --- | --- | --- |
| AtFtsZ2_FL_-mCerulean | | -45.0469 | 0.0012259 | 0.9988 | 0.083472 | 1.12E-05 | 11% |
| AtFtsZ1_FL_-mCerulean | | 0.3878 | 0.1994 | 0.8008 | 0.089883 | 0.0011147 | 19% |
| AtFtsZ2_C_-mCerulean | | 0.699 | 0.8897 | 0.1108 | 0.0049053 | 0.1158 | 17% |
| AtFtsZ1_C_-mCerulean | | 0.683 | 0.1689 | 0.831 | 0.401 | 0.012341 | 27% |
| AtFtsZ2_FL_-eYFP-MTS | | -4.9968 | 0.015308 | 0.9846 | 0.065542 | 1.93E-05 | 9.5% |
| AtFtsZ2_C_-eYFP-MTS | | -77.186 | 0.00057774 | 0.9994 | 0.1145 | 4.22E-06 | 8.7% |
| AtFtsZ1_FL_-eYFP-MTS | | 0.8492 | 0.5579 | 0.4354 | 0.086303 | 0.015592 | 14% |
| Z1_NT_Z2 _C_Z1_CT_-mCerulean | | 0.4984 | 0.055527 | 0.9427 | 0.9447 | 1.37E-03 | 10% |
| Z2_NT_Z1_C_Z2_CT_-mCerulean | | 0.5105 | 0.045257 | 0.9136 | 0.7871 | 2.04E-03 | 13% |
| GsFtsZA_FL_-mCerulean | | -0.1297 | 0.1097 | 0.8927 | 0.066348 | 0.0018657 | 32% |
| GsFtsZB_FL_-mCerulean | | -0.1044 | 0.1609 | 0.8374 | 0.021776 | 0.002693 | 46% |
| GsFtsZA_C_-mCerulean | | 0.6706 | 0.2035 | 0.7926 | 0.06294 | 0.0030585 | 18% |
| GsFtsZB_C_-mCerulean | | -16.1092 | 0.014844 | 0.9851 | 0.017144 | 4.47E-05 | 35% |
| GsFtsZA_FL_-eYFP-MTS | | -52.9617 | 0.0013873 | 0.9986 | 0.054183 | 5.53E-06 | 12% |
| GsFtsZA_C_-eYFP-MTS | | 0.8752 | 0.2412 | 0.7594 | 0.1703 | 0.013001 | 11% |
|  |  | |  |  |  |  |  |

Table S2. FRAP Parameters in Coexpression Strains.

| Proteins | *r* | | *C_eq1_* | | *C_eq2_* | | *k_off1_* | | *k_off2_* | | R_130_ | |
| --- | --- | --- | --- | --- | --- | --- | --- | --- | --- | --- | --- | --- |
| AtFtsZ2_FL_-eYFP-MTS  +  AtFtsZ1_FL_-mCerulean | -7.5032 | 0.010263 | | 0.9897 | | 0.065403 | | 3.55E-05 | | 13% | |  |
|  | 0.6694 | 0.4819 | | 0.517 | | 0.0054528 | | 0.1471 | | 26% | |  |
| AtFtsZ2_C_-eYFP-MTS  +  AtFtsZ1_C_-mCerulean | 0.8847 | 0.4323 | | 0.5641 | | 0.08024 | | 0.018887 | | 11% | |  |
|  | 0.5786 | 0.3805 | | 0.614 | | 0.022995 | | 0.006471 | | 31% | |  |
| GsFtsZA_FL_-mCerulean  +  GsFtsZB_FL_-eYFP | 0.3254 | 0.0011476 | | 0.9587 | | 1.8425 | | 0.0075659 | | 42% | |  |
|  | 0.5874 | 0.0014012 | | 0.9266 | | 0.4577 | | 0.014479 | | 35% | |  |
| GsFtsZA_FL_-mCerulean  +  GsFtsZB_C_-eYFP | 0.4145 | 0.1421 | | 0.856 | | 0.053533 | | 0.0091726 | | 44% | |  |
|  | -281.7766 | 0.001349 | | 0.9986 | | 0.012668 | | 1.67E-06 | | 39% | |  |

Table S3. Primers Used in the Study.

| Name | Sequences |
| --- | --- |
| LY33 | CTAATTATTCGAAACGAGGAATTCATGCATCATCATCATCATCATGCTTCTGATTCTGTGTCATCTCCTCC |
| LY34 | CTCGCCCTTGCTCACCATTCTAGACTTGTTCTCTTGCTGGAACCTTCTAAGG |
| LY242 | TCGAAACGAGGAATTCATGCATCATCATCATCATCATCCTTGCATTATCAAGGTTGTGGG |
| LY244 | CTTGCTCACCATTCTAGAAGGAAATCCAGTAGCGATCACGG |
| LY36 | CTAATTATTCGAAACGAGGAATTCATGCATCATCATCATCATCATGATTCTACTACTACCACCACTACAACCAC |
| LY37 | CTCGCCCTTGCTCACCATTCTAGAAGAGAATCCGGTAGCCACCACAGTAAC |
| LY243 | TCGAAACGAGGAATTCATGCATCATCATCATCATCATCAGTGCAAGATTAAGGTTGTGGG |
| LY246 | TGCTCACCATTCTAGAAGAGAATCCGGTAGCCACCACA |
| LY330 | AATTATTCGAAACGAGGAATTCATGCATCATCATCATCATCATGCGAGGATTAAGGTTATTGGTGTGG |
| LY334 | CTTGCTCACCATGGTACCGAAACCCGTAGCTATCAGGGTT |
| LY34 | CTCGCCCTTGCTCACCATTCTAGACTTGTTCTCTTGCTGGAACCTTCTAAGG |
| LY35 | CTCGCCCTTGCTCACCATGGTACCCTTGTTCTCTTGCTGGAACCTTCTAAGG |
| LY242 | TCGAAACGAGGAATTCATGCATCATCATCATCATCATCCTTGCATTATCAAGGTTGTGGG |
| LY340 | CAGGCTTCAAACGACAAGAAGAGGGAGAAGGAC |
| LY341 | TGCTCACCATTCTAGAGACTCGGGGATAACGAGAGCT |
| LY336 | TCCAATTGACAAGCTTTTGATTTTAACGACTTTTAACGACAACTTGAG |
| LY337 | ATTCTCGCCTCATTGTAGTTACTCGGAGCAGATGG |
| LY338 | ACAATGAGGCGAGAATTAAGGTGATTGGTGTCG |
| LY339 | TGTCGTTTGAAGCCTGTGGCGATTATCGTT |
| LY336 | TCCAATTGACAAGCTTTTGATTTTAACGACTTTTAACGACAACTTGAG |
| LY343 | ATCCTCGCAGATTCCATCGGAGAGAAGGAAC |
| LY344 | TGGAATCTGCGAGGATTAAGGTTATTGGTGTGG |
| LY345 | GACTGAGAGAAACCCGTAGCTATCAGGGTTATG |
| LY346 | CGGGTTTCTCTCAGTCATTCCAGAAGACACTTCTGAC |
| LY347 | TGCTCACCATTCTAGAGAAGAAAAGTCTACGGGGAGAAGACG |
| CC229 | AGGGATTTTGGTCATGAGATCGCTAGCAGATCTAACATCCAAAGACGAAAGGTTG |
| LY190 | CGTCTTTGGATGTTAGATCTTCTCACTTAATCTTCTGTACTCTGAAGAGGAG |
| LY201 | GTGGTGGCTACCGGATTCTC |
| LY328 | TGTCTAAGGCTAAAACTTTACTTGTACAGCTCGTCCATGCC |
| LY329 | AGTTTTAGCCTTAGACATGACTGTTCC |
| LY192 | CTATGGTGTGTGGGGGATCCTCTCACTTAATCTTCTGTACTCTGAAGAGGAG |
| LY410 | CGAGTTGTCTATCACCGTGATCG |
| LY328 | TGTCTAAGGCTAAAACTTTACTTGTACAGCTCGTCCATGCC |
| LY253 | GAAGGAGATATACATATGGCCGCTCAGAAATCTGAATCTT |
| LY252 | TTGTTAGCAGCCGGATCCTCAGTGGTGGTGGTGGTGGTGGACTCGGGGATAACGAGAGCT |
| LY251 | GAAGGAGATATACATATGAGGTCTAAGTCGATGCGATTGAGG |
| LY254 | GTTAGCAGCCGGATCCTCAGTGGTGGTGGTGGTGGTGGAAGAAAAGTCTACGGGGAGAAG |
| LY332 | AATTATTCGAAACGAGGAATTCATGCATCATCATCATCATCATGCGAGAATTAAGGTGATTGGTGTCG |
| LY335 | CTTGCTCACCATGGTACCGAAGCCTGTGGCGATTATCGT |
